# Sex-specific virtual population for the prediction and assessment of arrhythmia risk

**DOI:** 10.1093/europace/euag133

**Published:** 2026-05-29

**Authors:** Fengze Sui, Dasen Yan, Xiangbin Meng, Chengxiang Duan, Yucheng Zhang, Wen Gao, Zhen Song

**Affiliations:** School of Computer Science and Technology, Harbin Institute of Technology, HIT Campus, Shenzhen University Town, Nanshan District, Shenzhen 518055, Guangdong, China; Pengcheng Laboratory, No. 2 Xingke 1st Street, Nanshan District, Shenzhen 518000, Guangdong, China; Pengcheng Laboratory, No. 2 Xingke 1st Street, Nanshan District, Shenzhen 518000, Guangdong, China; Pengcheng Laboratory, No. 2 Xingke 1st Street, Nanshan District, Shenzhen 518000, Guangdong, China; Thomas Lord Department of Computer Science, Viterbi School of Engineering, University of Southern California, Los Angeles, CA 90089, USA; Pengcheng Laboratory, No. 2 Xingke 1st Street, Nanshan District, Shenzhen 518000, Guangdong, China; School of Computer Science and Technology, Harbin Institute of Technology, HIT Campus, Shenzhen University Town, Nanshan District, Shenzhen 518055, Guangdong, China; Pengcheng Laboratory, No. 2 Xingke 1st Street, Nanshan District, Shenzhen 518000, Guangdong, China; Pengcheng Laboratory, No. 2 Xingke 1st Street, Nanshan District, Shenzhen 518000, Guangdong, China

**Keywords:** Sex stratification, Sex hormones, Virtual population, In silico trial, Long QT syndrome, Drug-induced arrhythmia

## Abstract

**Aims:**

Women are at higher risk of serious ventricular arrhythmias, including torsades de pointes, when repolarization reserve is reduced, but sex-stratified mechanisms and quantitative risk assessment remain challenging. We aimed to develop scalable, tissue-scale, sex-aware ventricular virtual populations to quantify and explain sex differences in inherited and acquired proarrhythmic susceptibility.

**Methods and results:**

We constructed male and female virtual cohorts using a one-dimensional ventricular cable model with pseudo-electrocardiogram (pseudo-ECG), integrating sex-specific ionic conductance backgrounds and acute sex-hormone modulation. Virtual populations were filtered under multi-condition stress tests and calibrated to clinical corrected QT interval (QTc) distributions. We generated long QT syndrome types 1–3 (LQT1–3) cohorts, simulated sympathetic stress, and performed virtual drug trials for 109 compounds using multichannel block profiles at 1× effective free therapeutic plasma concentration. Proarrhythmic risk was defined by tissue-scale instability events (premature ventricular complexes, T-wave alternans, or repolarization failure). Drivers were analysed using regression and channel-sensitivity analysis, and clinical 24 h concentration-ECG data after dosing were used for external validation. The female cohorts exhibited higher simulated event risk across long QT syndrome subtypes and across multichannel drug block profiles, including drugs with <10 ms mean QTc prolongation. Female-to-male risk ratios tracked clinical risk categories. Simulated QTc time-courses and concentration-QTc trends agreed with 24 h clinical ECG data for key reference drugs.

**Conclusion:**

Sex-aware tissue-scale virtual populations enable in silico trials that quantify proarrhythmic risk beyond mean ΔQTc, provide mechanistic drivers, and support sex-informed cardiac safety evaluation and monitoring strategies.

## Graphical Abstract

**Graphical Abstract:**
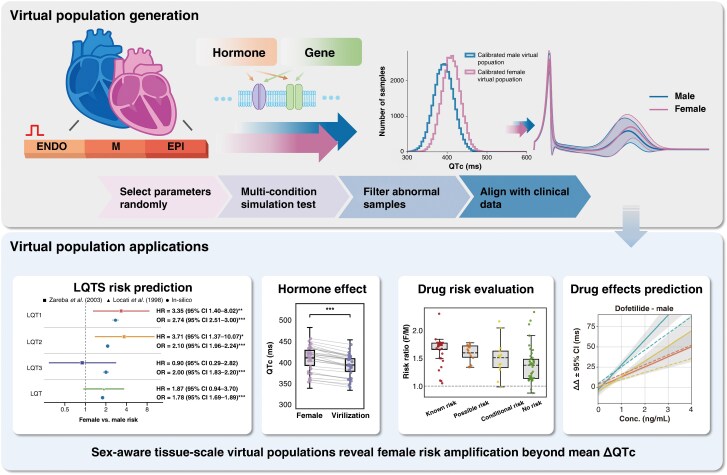

Translational perspective Current clinical and regulatory practice often emphasizes mean QTc prolongation, yet women may experience disproportionate proarrhythmic risk when repolarization reserve is impaired. By integrating sex-specific ionic backgrounds, acute hormone modulation, genetic vulnerability (LQT1–3), sympathetic stress, and multichannel pharmacology into tissue-scale virtual populations, our in silico trials provide sex-stratified risk curves and identify mechanistic drivers (e.g. enhanced female susceptibility to *I*_Kr_ block). This framework can complement existing ECG-based surveillance and model-informed drug development by prioritizing sex-informed monitoring and dosing strategies, including for drugs with small average ΔQTc but amplified risk in vulnerable subgroups.

## Introduction

Sex is a key biological variable shaping ventricular repolarization and susceptibility to ventricular arrhythmias. At rest, women typically have longer corrected QT (QTc) and JT-related intervals than men.^1,2^ When repolarization reserve is reduced, such as QT-prolonging drugs, bradycardia, or electrolyte disturbances, women have a higher incidence of torsades de pointes (TdP) and other severe ventricular arrhythmias,^3–7^ and female sex is an independent risk factor in settings such as acquired atrioventricular block.^8^ Clinical consensus statements and safety guidelines therefore recommend considering sex in monitoring and in cardiac safety evaluation.^4,6^

Despite this recognition, actionable sex-stratified risk quantification remains difficult. QT/QTc prolongation is the dominant biomarker in clinical and regulatory decision-making, yet measurement heterogeneity and heart-rate correction methods can yield inconsistent sex effects across cohorts.^9,10^ Moreover, TdP can occur even in patients with basal QTc values in the ‘normal’ range.^11,12^ In drug development, a ‘negative’ thorough QT/QTc (TQT) study can rule out a mean QTc effect in healthy volunteers, but cannot exclude meaningful risk amplification in susceptible subpopulations with impaired repolarization reserve.

Mechanistically, sex differences reflect interacting determinants across scales. Sex-related differences in ventricular ion-channel expression can alter the magnitude and transmural gradients of repolarizing currents.^13^ Sex steroid hormones further modulate ion channels acutely (non-genomic effects on conductance and kinetics^14–16^) and chronically (receptor-mediated trafficking and expression remodelling^17^). Clinically, QT sex differences emerge after puberty, whereas congenital long QT syndrome (LQTS) cohorts suggest smaller pre-pubertal sex differences in event risk,^18–22^ underscoring dynamic and individual variability.

Population-based computational electrophysiology offers a controlled route to integrate these determinants and to perform mechanism inference and counterfactual analyses.^23,24^ Prior *in silico* studies of drug-induced proarrhythmia span complementary levels of model complexity. Cell-scale population-of-models studies and sex-specific myocyte or machine-learning frameworks have improved prediction of torsadogenic liability and identified sex-dependent electrophysiological signatures,^25,26^ yet they rely on cellular surrogates and cannot represent propagated tissue or ECG behaviour.^27^ Three-dimensional ventricular and torso simulations, including recent sex-specific cardiac emulators, can reproduce body-surface ECG biomarkers under multichannel drug block,^28^ but their computational cost limits large sex-stratified virtual cohorts and systematic drug screening. *In silico* trial studies evaluating treatment stratification^29^ have further highlighted the translational value of virtual-patient cohorts for electrophysiological screening.

One-dimensional (1D) cables offer an intermediate scale that balances these considerations, although they cannot sustain re-entry and may not preserve all repolarization heterogeneities present in 2D or 3D tissue, they can capture selected propagated repolarization-instability endpoints and pseudo-ECG behaviour at a throughput compatible with large virtual cohorts. We therefore use a sex-aware 1D ventricular cable framework as a complementary approach, designed for mechanistic population-scale evaluation of sex, genotype, sympathetic stress, and multichannel drug block.

Here we combine sex-specific ionic conductance backgrounds (from reported human ventricular expression differences^13^) with acute sex-hormone modulation to construct male and female virtual populations calibrated to clinical QTc distributions and to generate LQT1–3 cohorts. We then perform virtual stress tests under sympathetic stimulation and multichannel drug block for 109 drugs, quantify sex-specific risk amplification using tissue-scale events, identify key drivers via regression and channel-sensitivity analysis, and evaluate transferability using time-resolved 24 h clinical concentration-ECG data.

## Methods

This study was reported using the European Heart Rhythm Association (EHRA) AI checklist,^30^ and the completed EHRA AI Checklist is provided as Supplementary material. No new human or animal experiments were performed. External clinical datasets used for calibration and validation were obtained from publicly available sources cited in the manuscript.

### Model overview and pseudo-ECG computation

In this study, we choose the ToR-ORd human ventricular action potential (AP) model,^31^ and apply 1D cable model to generate pseudo-ECG. The voltage equation and ECG compute equation of 1D cable are as follows:


$$\begin{array}{c} \frac{\partial V_{m}}{\partial t}=-\frac{I_{\mathrm{ion}}+I_{\mathrm{stim}}}{C_{m}}+\nabla \cdot (D\nabla V) \end{array}$$

Where *V* is the membrane potential, *C*_m_ = 1 μF/cm^2^ is the membrane capacitance, *I*_ion_ denotes the total ionic current density, *I*_stim_ = −53 μA/cm^2^ is the stimulus current applied for 1 ms to the first five endocardial cells at the start of each pacing cycle length (PCL), and *D* = 1.11 × 10^−4^ cm^2^/ms is the diffusion coefficient. The pseudo-ECG was calculated using


$$\begin{array}{c} \mathrm{ECG}=\int D\nabla V_{m}\cdot \nabla \left(\frac{1}{r}\right)dx \end{array}$$

where *r* = $\sqrt{{\left(x-x_{e}\right)}^{2}+{\left(y-y_{e}\right)}^{2}}$ represents the Euclidean distance from each point (*x*, *y*) on the cable to the virtual electrode at (*x_e_*, *y_e_*). The electrode position is defined as follows: *x_e_* = 15 × 0.004 cm = 0.06 cm, corresponding to the 15th node along the cable; *y_e_* = 0.2 cm, representing the perpendicular distance from the cable, consistent with typical clinical ECG lead configurations. The simulations employed a variable time step ranging between 0.01 and 0.1 ms, and output data were sampled at 1 ms intervals to facilitate efficient post-processing. Euler integration was applied to compute single-cell ionic currents and intercellular diffusion of electrical signals.

**Figure 1:**
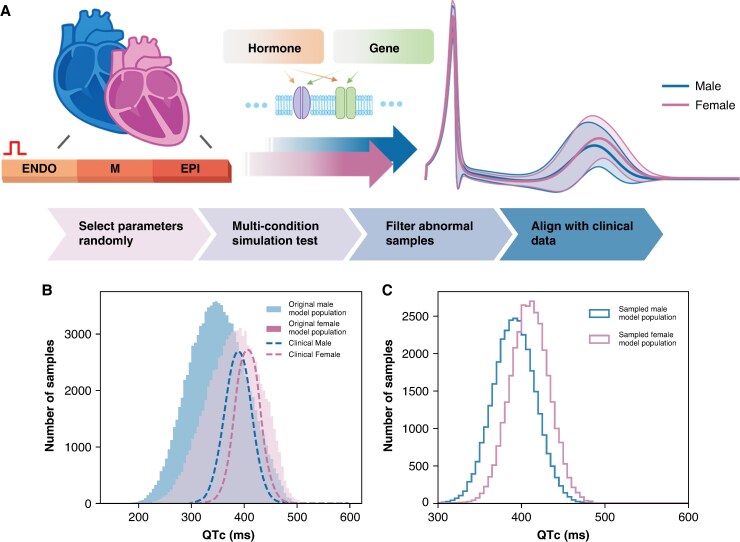
Workflow for constructing the virtual populations and the QTc distributions. (*A*) Overview of the population generation pipeline. Each virtual subject is represented by a 1D ventricular cable model comprising Endo, M, and Epi cells. Model parameters are modulated by sex hormones and sex-specific ion-channel expression. After sequential filtering, the final virtual populations are obtained. The right panel shows the mean pseudo-ECG waveforms and standard deviations (SDs) for the final male and female populations. (*B*) QTc distributions of the raw virtual populations and the reference clinical QTc distributions (dashed lines). The clinical male QTc distribution was modelled as a Gaussian with *μ* = 388.45 ms and *σ* = 25.52 ms, and the clinical female distribution with *μ* = 407.01 ms and *σ* = 23.38 ms. (*C*) QTc distributions of the male and female virtual populations were obtained by distribution-matched sampling from the raw populations.

The 1D fibre comprised 304 nodes (spatial step of Δx = 0.004 cm, total length ∼1.22 cm, consistent with human ventricular wall thickness), divided into three regions to reflect transmural heterogeneity. The endocardial (Endo) region included nodes numbered 225–304. The midmyocardial (M) region encompassed nodes numbered 90–224. The epicardial (Epi) region covered nodes numbered 1–89 (*Figure 1A*).

### Sex-specific ionic conductance backgrounds

Sex-related genetic differences were represented as sex-specific differences in ionic maximal conductances. To define plausible ranges for sex-related variation, we followed Gaborit et al.,^13^ who quantified sex differences in ion-channel subunit expression in non-diseased human ventricular epicardial and endocardial tissue, and we ensured that our initial ranges encompassed those reported in other sex-specific population modelling studies.^32–34^ Parameter ranges are summarized in Supplementary material online, *Table S1*.

### Sex hormones effect

Following Yang et al.,^35^ the acute (non-genomic) effects of sex hormones were incorporated by scaling the maximal conductance of selected ionic currents with multiplicative factors, while keeping the original gating kinetics unchanged. Specifically, for a current *I_x_*, the maximal conductance was updated as


$$\begin{array}{c} G_{x}=G_{x}^{\left(0\right)}\cdot S_{x}(C) \end{array}$$

where $G_{x}^{\left(0\right)}$ is the baseline conductance, and $S_{x}(C)$ is a Hill-type saturating function of the hormone concentration *C* (in nM). The functional forms and parameter values were from the cell membrane patch clamp experiments.^14,36,37^

Testosterone enhances slow delayed rectifier potassium current (*I*_Ks_):


$$\begin{array}{c} S_{\mathrm{Ks}}(T)=1+0.4\frac{T}{0.7+T}\; \end{array}$$

Testosterone inhibits L-type calcium current (*I*_Ca,L_):


$$\begin{array}{c} S_{\mathrm{Ca},L}(T)=1-\;0.2\frac{T^{2}}{15^{2}+T^{2}}\; \end{array}$$

Progesterone enhances *I*_Ks_:


$$\begin{array}{c} S_{\mathrm{Ks}}(P4)=1+0.4\frac{P4}{2.6+P4}\; \end{array}$$

Estradiol inhibits rapid delayed rectifier potassium current (*I*_Kr_):


$$\begin{array}{c} S_{\mathrm{Kr}}(E2)=1-0.19\frac{E2}{0.6+E2}\; \end{array}$$

T, P4, and E2 denote testosterone, progesterone, and estradiol concentrations (nM), respectively. The hormone concentration ranges used for each sex are provided in the Supplementary material online, *Table S2*.

### Generation of the virtual population

Random parameter sets were sampled from pre-assigned experimental intervals (see Supplementary material online, *Tables S1* and *S2*) to generate candidate virtual subjects. Each candidate parameter set was then subjected to a sequential, multistage filtering pipeline using 1D cable simulation to ensure physiological baseline behaviour and tissue-level electrical stability. For each randomly drawn parameter set, we applied the following stage-wise screening procedure.

Stage 1: baseline screening (PCL = 1 s). Each candidate parameter set was paced for five beats in total. The first four beats were used to allow initial transients to dissipate, and the 5th beat was used for baseline metric extraction. Parameter sets were discarded if AP or calcium-cycling properties were abnormal at the single-cell level, if cable-level instability such as premature ventricular complexes (PVCs), T-wave alternans (TWA), or repolarization failure (RF) was observed during the baseline run, or if QRS duration fell outside 60–100 ms.

Stage 2: sympathetic-stress screening (PCL = 1 s). Sympathetic stress was modelled by doubling *I*_Ca,L_ amplitude. Each candidate parameter set was paced for 10 beats in total under this condition. The first 6 beats served as settling beats, and the subsequent 4 beats were used for evaluation. Parameter sets that developed PVCs, TWA, or RF during this stage were excluded.

Stage 3: rate-response screening (PCL = 0.4 and 1.5 s). For candidates that passed Stage 2, additional pacing at PCL = 0.4 s and PCL = 1.5 s was performed to evaluate fast and slow rate responses. At each PCL, 10 beats were simulated in total, and the first 6 beats served as settling beats, and the subsequent 4 beats were used for evaluation. Parameter sets that developed PVCs, TWA, or RF were excluded.

Stage 4: confirmatory stability screening (PCL = 1 s). Candidates were then returned to PCL = 1 s for a final stability check. Again, 10 beats were simulated in total, with the first 6 beats treated as settling beats and the following 4 beats used for evaluation. Candidates exhibiting PVCs, TWA, or RF during this evaluation window were excluded.

By iterating the above pipeline, we obtained the original male and female populations. The subsequent LQT and drug simulations adopted the same prepacing settings as Stages 2, 3, and 4 (10 beats total, 6 settling beats followed by 4 evaluation beats). Detailed single-cell and cable-level filtering criteria, together with representative examples of PVC, TWA, and RF, are provided in the Supplementary Methods and Supplementary material online, *Figure S7*.

### LQTS virtual populations

To construct disease populations for LQTS (LQT1, LQT2, and LQT3), we modified the conductance of specific ionic currents. Specifically, we reduced the maximal conductance of *I*_Ks_ (*G*_Ks_) to model LQT1, reduced maximal conductance of *I*_Kr_ (*G*_Kr_) to model LQT2, and increased maximal conductance of the late sodium current (*I*_Na,L_, denoted *G*_Na,L_) to model LQT3. The scaling range for *G*_Ks_ was 0.15–0.65, and for *G*_Na,L_ was 1.2–1.7. For *G*_Kr_, the adjusted range varies slightly between male and female groups. For male, the range is between 0.4 and 0.9, while for female, it is slightly lower, ranging from 0.35 to 0.85. For each virtual subject, we randomly sampled a scaling factor from the corresponding range and performed simulations with a PCL of 1 s, excluding individual models who exhibited PVCs, TWA, or RF, to obtain the LQTS virtual populations.

### Drug virtual trials and multichannel block implementation

We evaluated 109 drugs using sex-specific normal virtual populations. Drug effects on ion channels were parameterized by half-maximal inhibitory concentration (IC_50_) and Hill coefficients, and drug exposure was represented by effective free therapeutic plasma concentrations (EFTPC), consistent with our previous work.^38^ Simulations were performed at 1× EFTPC, and channel conductances were scaled using concentration–effect relationships to represent multichannel block. Drug effects were modelled using a simple pore block approach, adjusting ion channel conductance via the Hill equation:


$$\begin{array}{c} G_{\mathrm{ion}}^{\prime }=G_{\mathrm{ion}}{\left[1+{\left(\frac{D}{IC_{50,\mathrm{drug}}}\right)}^{h}\right]}^{-1} \end{array}$$

where *G*_ion_ is the original conductance, *D* is the EFTPC, IC_50,drug_ is the half-maximal inhibitory concentration, and *h* is the Hill coefficient.

### Risk estimation, risk ratios, and statistical analyses

For each drug and sex, event incidence (risk) was computed as the fraction of individual models with any arrhythmic event. To quantify sex differences, we aggregated drugs by clinical risk category and computed the female-to-male risk ratio. Because event rates can be extremely low, we applied Jeffreys prior smoothing (Beta(0.5, 0.5)) to event rates before calculating risk ratios.

For LQTS cohorts, sex effects were quantified using a binary logistic regression model within each subtype, using males as the reference group. Sex-specific risk was reported as the odds ratio (OR) for females vs. males with 95% confidence intervals from the Wald method.

To quantify the electrophysiological drivers of sex differences in proarrhythmic risk across multichannel block profiles, we used the probability of arrhythmic events for each of the 109 drugs in the male and female virtual populations as the outcome and applied a *y* = log(1 + risk) transform to reduce skewness and improve linear interpretability. After stacking male and female samples, we fitted a ridge regression model that included both main effects and interaction terms $\mathrm{sex}\times \mathrm{in}h_{k}$ to assess sex modification of channel effects:


$$\begin{array}{c} y=\alpha +\beta_{\mathrm{sex}}\mathrm{sex}+\underset{k}{\sum }\;\beta_{k}\mathrm{in}h_{k}+\underset{k}{\sum }\;\gamma_{k}(\mathrm{sex}\cdot \mathrm{in}h_{k})\; \end{array}$$

Here, $\mathrm{in}h_{k}$ denotes the inhibition fraction (0–1) for ion channel *k*.

Channel-sensitivity analyses were performed by increasing one channel’s inhibition fraction from its 5th to 95th empirical percentile while holding others constant, and comparing predicted changes in log(1 + risk).

### External validation with time-resolved clinical concentration-ECG data

To evaluate transferability, we extracted plasma drug concentrations and time-resolved changes in QTc, the heart rate-corrected J-T_peak_ interval (J-T_peak_c), and the interval from T-wave peak to T-wave end (T_peak_–T_end_) within 24 h after dosing from clinical studies in healthy volunteers stratified by sex. These biomarkers were selected because they capture complementary aspects of repolarization for safety pharmacology: QTc indexes overall ventricular repolarization delay, J-T_peak_c reflects the early phase of repolarization and is sensitive to the balance among ion-channel contributions, and T_peak_–T_end_ captures transmural and spatial dispersion of late repolarization.

**Figure 2:**
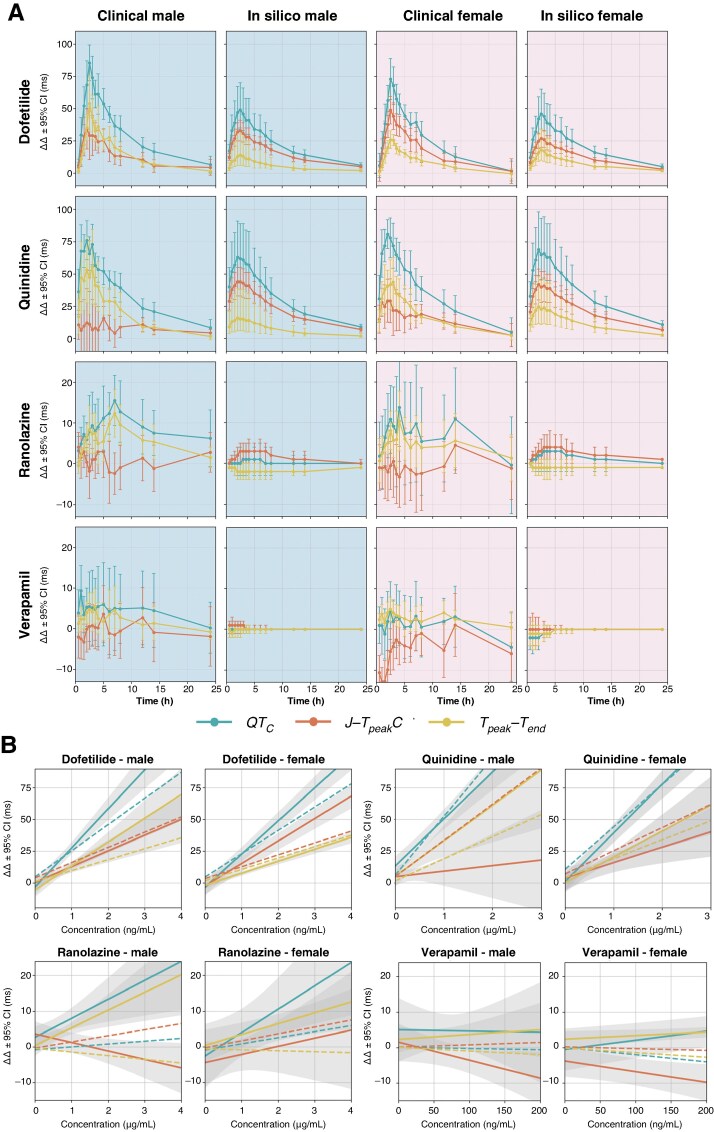
Evaluation of the consistency between clinical and simulated ECG feature changes over time and concentration after dosing. (*A*) Time courses of ECG feature changes within 24 h after dosing for four drugs, comparing clinical observations and simulation predictions. The column directions are presented in the following sequence (left to right): clinical males, simulated males, clinical females, and simulated females. Rows (top to bottom): dofetilide, quinidine, ranolazine, and verapamil. The *y*-axis shows feature changes (ΔΔ), the error bars in the clinical data indicate 95% confidence intervals, and the *x*-axis shows time after dosing. Simulated trajectories are summarized by the population median, and the vertical bars denote the pointwise 25th–75th percentile across virtual individuals. (*B*) Concentration–effect relationships for ECG features, comparing clinical data (solid lines) and simulations (dashed lines). The clinical curves are fitted from subject-level ΔΔ data, whereas the dashed simulated lines are linear fits to population-mean simulated responses at each concentration-time point. Grey shading denotes 95% confidence intervals for the clinical fits. Distributions of subject-specific simulated slopes are shown in Supplementary material online, *Figure S6*.

In simulations, plasma concentration was combined with a fixed unbound fraction (dofetilide, 0.357; quinidine, 0.15; ranolazine, 0.321; verapamil, 0.157)^16^ to obtain free concentration and map each time point to channel inhibition, after which ECG biomarker changes were simulated in the normal virtual populations. More specifically, at each clinical time point, total plasma concentration was multiplied by the corresponding fixed unbound fraction to estimate free concentration, channel-specific inhibition fractions were then computed from the Hill equation (Eq. 8), the affected channel conductances were updated in each virtual subject, and QTc, J-T_peak_c, and T_peak_–T_end_ were recalculated from the simulated pseudo-ECG. For illustration, dofetilide at its peak clinical concentration (∼2.5 nM total) yields a free concentration of ∼0.89 nM (fu = 0.357), producing ∼48% *I*_Kr_ inhibition with minor Ito inhibition (∼8%) and negligible effects on other channels (<0.01%), which drives the pronounced QTc prolongation seen in *Figure 2A*. QTc and J-T_peak_c were corrected using Fridericia’s formula.^39^

For the clinical data, triplicate ECG measurements were first averaged within each unique combination of subject, period, treatment, and time point, and concentration–effect relationships were then estimated from the resulting ΔΔ values using ordinary least-squares regression. For the simulations, biomarker changes were computed for all virtual subjects at each concentration-time point, and the dashed lines shown in *Figure 2B* were obtained by fitting linear regressions to the population-mean simulated responses across time points. Because these fits summarize average virtual-population behaviour, we additionally fitted subject-specific concentration–effect slopes for each virtual subject and summarized their distributions in Supplementary material online, Figure  *S6*.

## Results

### Construction of the virtual population

**Figure 3:**
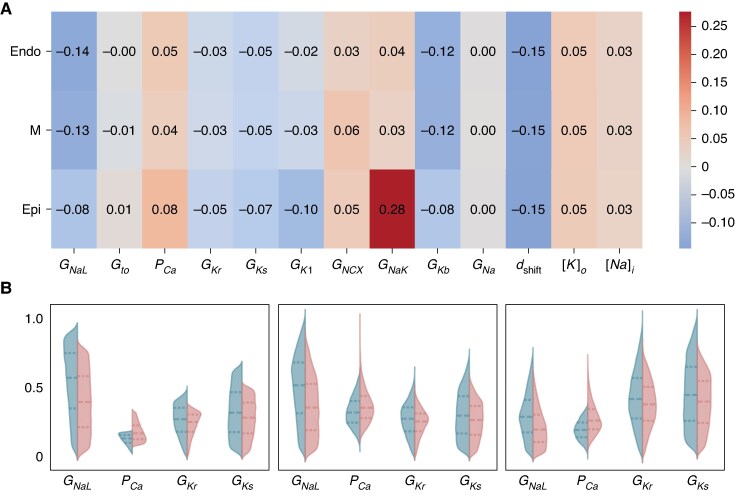
**Parameter distributions in the virtual populations.** (*A*) Heatmap of differences between female-specific and male-specific parameter means (female minus male). Red indicates higher parameter values in females, while blue indicates higher values in males. (*B*) Violin plots of four selected parameters across the three myocardial regions (Endo, M, and Epi). The left half of each violin denotes the male distribution and the right half denotes the female distribution.

To generate the virtual populations, we performed random sampling within the ranges of ion-channel and hormone-related parameters to create candidate parameter sets, followed by electrophysiological simulations and filtering under multiple conditions. Specifically, under baseline pacing, we evaluated cellular stability and excluded individual models exhibiting abnormal APs, calcium transients, or triggered activity. We then assessed stability under sympathetic stress and across different pacing cycle lengths, excluding individual models who developed PVCs, TWA, or RF, yielding an initial virtual population (*Figure 1B*). We subsequently performed distribution-matched sampling based on clinical QTc distributions in healthy men and women^40^ to construct normal male and female virtual populations with QTc distributions consistent with clinical data (*Figure 1C*). The final male population comprised 31 839 individual models with a QTc of 390.8 ± 27.5 ms (mean ± SD), and the final female population comprised 31 860 individual models with a QTc of 411.17 ± 26.6 ms. These populations were used as the normal virtual cohorts in subsequent experiments. The mean QTc differed by ∼20 ms between sexes, consistent with prior clinical reports.^41,42^ The overall parameter distributions are shown in *Figure 3*. To facilitate cross-parameter comparisons, the parameters were globally normalized, and full violin plots are provided in the Supplementary material online, *Figure S1*. Meanwhile, the conduction velocity of the cable and the calculation method are presented in Supplementary material online, *Figure S5* and the Supplementary Methods.

**Figure 4:**
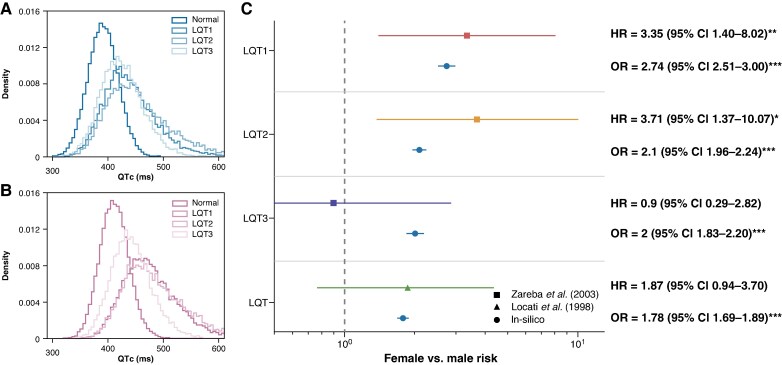
QTc distributions and risk prediction in sex-specific LQTS populations. (*A*) QTc distribution in the male LQTS populations. (*B*) QTc distribution in the female LQTS populations. (*C*) The ratio of risk of LQTS in the female group to that in the male group. The blue lines represent our simulation prediction results, white the other colours represent clinical data. The first three columns of clinical data (LQT1–3) are from the same study, and the last column (LQT, without subtype distinction) is from other studies. The grey dotted line is the reference line for a risk ratio of 1.

*Figures 4A* and *B* show the QTc distributions of these populations, which were close to the summary statistics reported by Vink et al.^40^ Numerous clinical studies have characterized QTc in LQTS cohorts,^43–45^ and reported sex-specific QTc distributions can vary across studies. Sometimes, they even show opposite trends, which is potentially due to differences in sample size and in QT/QTc calculation methods. To ensure consistency of the reference clinical data, we primarily compared our simulated distributions with those reported by Vink et al.

### Risk assessment in LQTS populations

After generating the LQTS virtual populations, we evaluated sex differences in arrhythmia risk. Sympathetic stimulation was modelled by doubling *I*_Ca,L_,^46^ and an event was considered to occur if PVCs, TWA, or RF were observed; otherwise, no event was recorded. All simulation results were summarized, with a total of 82 564 individual records included, among which 27 873 were LQT1, 23 386 were LQT2, and 31 305 were LQT3. The results showed that females had a higher risk in all three subtypes. In LQT1, the OR for females vs. males was 2.74 (95% confidence interval [CI], 2.51–3.00; *P* < 0.001). In LQT2, the OR was 2.10 (95% CI, 1.96–2.24; *P* < 0.001). In LQT3, the OR was 2.00 (95% CI, 1.83–2.20; *P* < 0.001). Further, when the three subtypes were combined and samples with a QTc less than 440 ms were excluded according to the inclusion criteria of clinical literature,^20^ the OR for females vs. males was 1.78 (95% CI, 1.69–1.89; *P* < 0.001).

For comparison, we juxtaposed our simulated estimates with metrics reported in previous clinical studies^20,21^ and summarized them in a forest plot (*Figure 4C*). Overall, our simulations produced sex-related risk differences in LQT1 and LQT2 that were consistent with clinical statistics. For LQT3, clinical studies did not observe a clear sex difference, whereas our simulations suggested higher risk in females. However, it should be emphasized that the simulation estimates still fell within the confidence intervals of the clinical studies. One possible explanation is that the number of LQT3 patients in the Zareba et al. cohort was substantially smaller than that of LQT1 and LQT2 patients (approximately half), leading to unstable effect estimates and greater statistical uncertainty. In contrast, Locati et al. did not stratify LQTS by genotype, but analysed an overall LQTS cohort with QTc > 440 ms. We adopted the same screening method, and the simulation prediction results were highly consistent with the clinical estimates.

To assess the robustness of these findings to key modelling assumptions, we performed two additional sensitivity analyses. Varying the M-region width by ±50% preserved the qualitative female-excess risk across LQT1–LQT3 and pooled LQTS (see Supplementary material online, *Figures S8 and S9*). A revised sympathetic-drive formulation with concurrent *I*_Ks_ and *I*_Ca,L_ enhancement attenuated QTc prolongation and absolute arrhythmic risk, but female-to-male ORs remained above 1 across all subtypes (see Supplementary material online, *Figures S10 and S11*). Together, these analyses indicate that the higher simulated arrhythmic risk in females is robust to plausible variations in structural assumptions and in the formulation of sympathetic drive.

### Effects of sex hormones on cardiac electrophysiology

**Figure 5:**
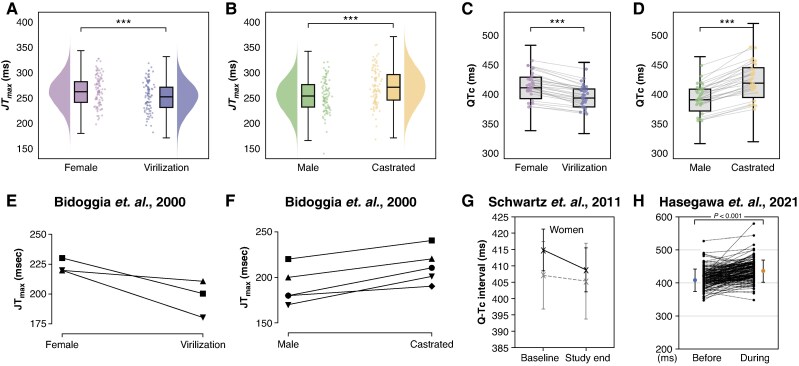
Changes in ECG features following hormone interventions in sex-specific populations. (*A–D*) Simulation results: distributions of JT_max_ before and after testosterone administration in females (*A*), JT_max_ before and after testosterone removal in males (*B*), QTc before and after testosterone administration in females (*C*), and QTc before and after testosterone removal in males (*D*). Panels A and B show 100 randomly sampled individual models and panels C and D show 30. Differences were assessed using two-sided t tests; ***P < 0.001. (*E–F*) Clinical findings reported by Bidoggia et al. (2000): shortening of JT_max_ in androgenized females (*E*) and prolongation of JT_max_ in castrated males (*F*). (*G*) Clinical QTc before and after transdermal testosterone therapy in women with chronic heart failure (Schwartz et al., 2011); the light grey dashed line indicates the placebo group. (*H*) Clinical QTc before and after androgen deprivation therapy in men (Hasegawa et al., 2021), showing significant QTc prolongation during treatment.

To verify whether the model captures hormonal modulation of repolarization, we applied an androgen switch: exogenous testosterone (35 nM) was administered to the normal female population and the testosterone effect was removed (0 nM) in the normal male population. Testosterone shortened the JT interval from the J point to the T-wave peak (JT_max_) and QTc in females, whereas androgen deprivation prolonged JT_max_ and QTc in males (*Figure 5A–D*). The direction of these changes agrees with clinical observations in androgen therapy and androgen deprivation studies,^47–50^ supporting population-level transferability of the hormone module.

### Drug-induced arrhythmia risk

**Figure 6:**
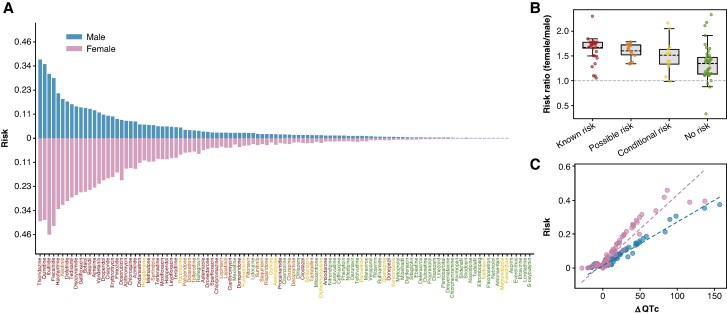
Drug-induced arrhythmia risk in normal male and female virtual populations. (*A*) Arrhythmic event risk for 109 drugs in the normal male and female virtual populations. Drugs are ordered by male risk from high to low. The *x*-axis lists drug names, and colours denote clinical risk categories: Known Risk (red), Possible Risk (orange), Conditional Risk (yellow), and No Risk (green). Bars indicate simulated risk in males (blue) and females (pink). (*B*) Female-to-male risk ratio aggregated by clinical risk category, and its association with risk category (Spearman *ρ* = −0.512, *P* = 1.263 × 10^−8^). (*C*) Relationship between drug-induced ΔQTc and event risk across the 109 drugs, with linear fits. Regression parameters in females: *k* = 0.00401, *b* = 0.0285 (*R*^2^ = 0.869); in males: *k* = 0.00258, *b* = 0.0137 (*R*^2^ = 0.951).

We systematically evaluated the arrhythmogenic risk of 109 drugs based on virtual populations of normal male and female. As in the analyses above, sympathetic stimulation was modelled by doubling *I*_Ca,L_ to increase arrhythmia susceptibility. Simulations were performed at 1× EFTPC, and channel conductances were scaled using the concentration–effect relationships to represent a multichannel block. For each individual, we recorded whether PVCs, TWA, or RF occurred, and the presence of any event was defined as an arrhythmic event. We then computed the event incidence in each sex-specific population (*Figure 6A*). Overall, the female population showed a higher post-dose event rate.

To further quantify and compare sex differences, we aggregated drugs by clinical risk category and computed the female-to-male risk ratio (*Figure 6B*). We encoded the clinical risk categories from Known Risk to No Risk as 1–4 and assessed the association between the risk ratio and risk category using Spearman's rank correlation. The correlation was negative (*ρ* = −0.512), indicating that the female-to-male risk ratio increased systematically as drugs moved towards higher clinical risk categories, i.e. women were more likely to experience arrhythmic events when exposed to clinically high-risk drugs.

We also quantified drug-induced QTc prolongation under baseline *I*_Ca,L_ (i.e. without sympathetic stimulation) and computed ΔQTc. When ΔQTc values for the 109 drugs were plotted against their corresponding event rates (*Figure 6C*), linear regression showed a steeper slope in females, implying that, for a given QTc change, the female population was more likely to develop arrhythmic events. These findings are consistent with clinical observations that sex itself is a major risk factor for drug-related arrhythmias, with multiple studies reporting a higher incidence of severe arrhythmias in women after drug exposure.^3–5^ For example, the clinical female-to-male risk ratio for ibutilide-induced TdP has been reported to be approximately 1.85,^51^ whereas our simulation yielded 1.50. Similarly, for sotalol, the reported clinical ratio was 2.16,^42^ whereas our simulation yielded 1.77; for dofetilide, the reported clinical ratio was 3.41,^52^ whereas our simulation yielded 1.71. Overall, the simulation results were consistent with clinical statistics in trend,^8^ and robustly reproduced the well-known phenomenon that women are more susceptible to drug-induced arrhythmic events. A sensitivity analysis in which sympathetic drive was modelled as concurrent 2.0*×I*_Ca,L_ + 2.0*×I*_Ks_ enhancement attenuated the absolute risk levels and the magnitude of inter-sex separation but did not alter the qualitative finding of higher female susceptibility (see Supplementary material online, *Figure S12*).

**Figure 7:**
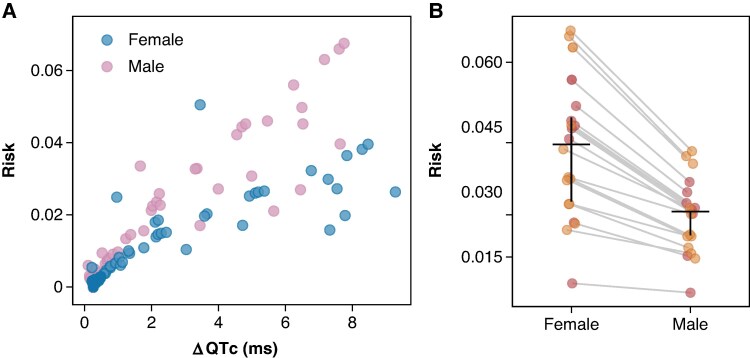
Sex differences for drugs with ΔQTc < 10 ms. (*A*) Scatter plot of QTc changes (upper bound < 10 ms) vs. event risk. (*B*) Sex differences in risk for drugs labelled as Known Risk and Possible Risk. Horizontal black lines denote medians, and vertical black lines show interquartile ranges. Paired Wilcoxon test, *P* < 0.001.

Furthermore, following the International Council for Harmonisation (ICH) E14 criterion for a ‘negative thorough QT/QTc (TQT)’ study, we identified drugs whose upper bound of the one-sided 95% CI for ΔQTc was <10 ms (*n* = 60; *Figure 7*). This threshold is commonly used in healthy volunteers to rule out an average QTc effect reaching regulatory concern.^53^ Although these 60 drugs produced relatively small QTc changes overall, arrhythmic risk still increased with QTc prolongation (Spearman *ρ*: female ≈ 0.94; male ≈ 0.93). Paired comparisons further showed that, for the same drug, female risk was systematically higher than male risk, with larger differences among drugs labelled as Known Risk or Possible Risk (*Figure 7B*). This finding is in line with the description of the boundary of the purpose of TQT studies in ICH E14. The aim of TQT is to determine whether there is a QT signal and to guide subsequent more cautious assessment in the target patient population. Prior work has also demonstrated that QTc as a biomarker can be dissociated from TdP risk.^54^ Even if the average effect of QTc is not significant, the risk may still be amplified in susceptible populations. In other acquired TdP settings, increased female risk is not fully explained by longer QT intervals. To minimize confounding by baseline QTc differences, we selected individual models from the overlapping QTc range between male and female populations, yielding sex-specific sub-cohorts with identical QTc distributions but substantially different genetic and hormonal backgrounds (see Supplementary material online, *Figure S3*). Repeating the drug risk experiments in these matched sub-cohorts produced essentially the same conclusions (see Supplementary material online, *Figure S2*).

### Analysis of sex difference

**Figure 8:**
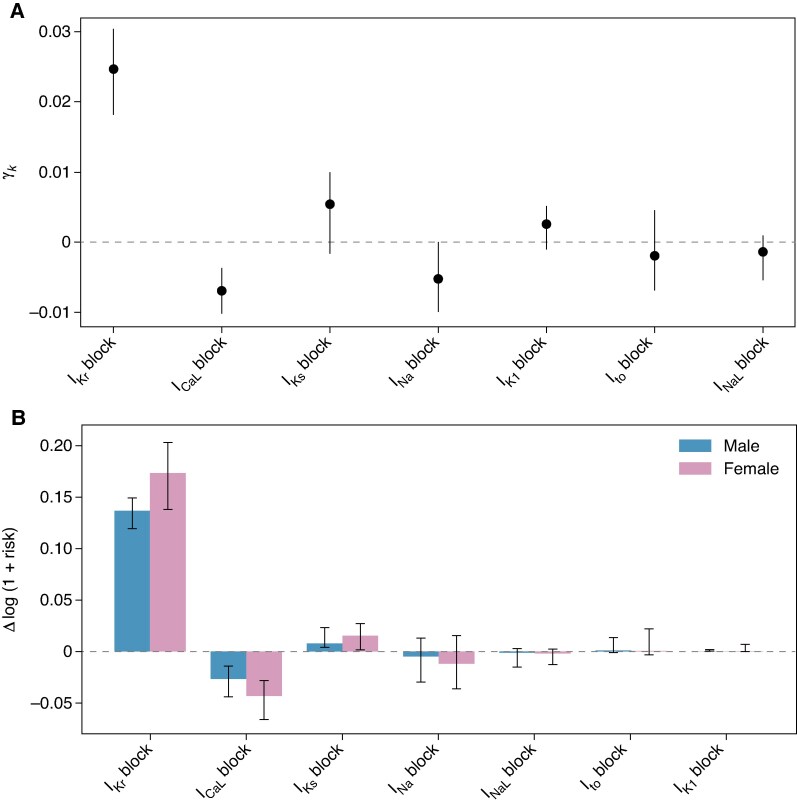
Sex-specific risk drivers and channel-sensitivity differences under multichannel block. (*A*) Interaction coefficients $\gamma_{k}$ from the ridge regression interaction model, which included sex, main effects of channel inhibition, and sex-by-channel interaction terms. The response was log(1 + risk), where risk is the probability of arrhythmic events for each drug in the corresponding sex-specific population. Points indicate bootstrap medians and error bars show bootstrap 95% confidence intervals. Positive $\gamma_{k}$ indicate that the channel block produces a larger risk increase in females than in males, while a negative $\gamma_{k}$ indicate that the channel block tends to be more risk-reducing in females. (*B*) Channel-sensitivity analysis within the observed domain. Bar heights represent bootstrap medians and error bars show bootstrap 95% confidence intervals.

The interaction terms showed a significantly positive coefficient for *I*_Kr_ block, indicating that, for the same degree of *I*_Kr_ inhibition, females exhibited higher predicted risk than males. In contrast, the interaction coefficient for *I*_Ca,L_ block was significantly negative, suggesting that *I*_Ca,L_ inhibition tended to reduce risk more strongly in females (*Figure 8A*). Channel-sensitivity analyses indicated that *I*_Kr_ remained the dominant risk driver in both sexes, with a larger effect magnitude in females. Conversely, *I*_Ca,L_ exhibited a negative (risk-reducing) effect, again stronger in females, whereas *I*_Ks_ had a secondary positive contribution that was more pronounced in females. Of note, the peak sodium current (*I*_Na_)-related signal in *Figure 8* appeared more prominent than *I*_Na,L_-related signal, this likely reflects the influence of peak *I*_Na_ on tissue excitability and conduction within the empirical inhibition range of the present drug panel, rather than a general mechanistic ranking of peak vs. late sodium current contributions to proarrhythmia.

Overall, the higher risk in females appeared to reflect greater sensitivity to perturbations of key repolarization-related channels coupled with stronger calcium pathway modulation, rather than being driven solely by baseline QTc differences. We additionally conducted counterfactual analyses across hormone states to explore potential mechanistic contributions. However, the results suggested pronounced non-additive interactions between hormone effects and ion-channel parameter backgrounds, making quantitative attribution sensitive to model specification. These analyses are therefore discussed in the Supplementary material online, *Figure S4*.

### Prediction of ECG repolarization biomarkers

To evaluate whether the constructed virtual populations can capture drug effects on ECG repolarization biomarkers, we extracted plasma drug concentrations and time-resolved changes in QTc, J-T_peak_c, and T_peak_–T_end_ over the 24 h after dosing from clinical studies in healthy volunteers, stratified by sex.^55,56^ For dofetilide and quinidine, both clinical data and simulations showed marked increases in repolarization biomarkers (*Figure 2A*). The simulated QTc time courses and overall magnitudes matched the clinical trends in both sexes, including the timing of peak effects and subsequent recovery. In contrast, predictions of J-T_peak_c and T_peak_–T_end_ deviated from clinical data in some cases. For ranolazine and verapamil, the clinically observed ECG changes were small and highly variable, whereas simulations showed weaker or near-zero effects.

To further assess model transferability, we paired ΔΔ values at each time point with the corresponding plasma drug concentrations and fitted concentration–effect relationships for both clinical and simulated data (*Figure 2B*). Overall, the QTc concentration–effect slopes for dofetilide and quinidine were in good agreement between simulations and clinical data in both sexes, although the simulated QTc slope for dofetilide was slightly lower than observed clinically. Larger discrepancies were observed for the decomposed metrics. Among males receiving quinidine, the clinical slope for J-T_peak_c was relatively small, whereas simulations predicted a steeper increase. Conversely, the simulated slope for T_peak_–T_end_ was lower in males. For ranolazine and verapamil, simulations more clearly underestimated the concentration dependence of QTc and the decomposed metrics, and in some instances the simulated trends differed from the clinical fitted trends.

Because the simulated slopes in *Figure 2B* summarize population-mean behaviour, we additionally examined subject-specific concentration–effect slopes (see Supplementary material online, *Figure S6*). Dofetilide and quinidine showed broadly consistent directions across most models. Ranolazine showed broader J-T_peak_c and T_peak_–T_end_ slope distributions concentrated closer to zero, whereas verapamil showed very narrow slope distributions centred near zero, consistent with minimal simulated concentration dependence.

## Discussion

Building on our previous framework, we incorporated sex as a biological variable by combining sex-specific ion-channel expression and acute sex-steroid modulation to construct matched male and female ventricular virtual populations, together with LQT1–3 cohorts. Across sympathetic stress and multichannel drug block, the platform quantified tissue-scale proarrhythmic events and reproduced the direction of repolarization changes with androgen manipulation. It also predicted sex-stratified, time-resolved QTc responses after dosing, supporting model transferability. This conclusion was also robust to a sensitivity analysis in which sympathetic drive was represented by concurrent enhancement of *I*_Ca,L_ and *I*_Ks_, which attenuated absolute QTc prolongation and arrhythmic risk but preserved the female excess risk across LQTS subtypes (see Supplementary Results and Supplementary material online, *Figures S10 and S11*).

Extensive clinical and pharmacovigilance evidence consistently demonstrates that females exhibit a heightened susceptibility to repolarization-related adverse cardiac events, particularly under conditions of QT interval prolongation or diminished repolarization reserve.^6,57–59^ However, this sex disparity is often discussed only at the level of statistical association or average QT prolongation, leaving unresolved whether sex differences persist when men and women have comparable QTc values, and what mechanisms drive any residual differences. By systematically evaluating 109 drugs in sex-specific virtual populations, we reproduced the consensus clinical observation that overall risk is higher in women. Moreover, repeating the analyses in sex-matched sub-cohorts with identical QTc distributions supported the generality of this conclusion. Given our population-generation strategy and stress-testing scenarios, these results suggest that elevated female risk is not driven solely by baseline QTc differences, but more likely reflects the combined modulation of sex-specific ion-channel parameter backgrounds and sex hormones. Therefore, even if the QTc distribution of male and female groups is consistent, under conditions such as drug block and sympathetic stimulation that reduce repolarization reserve, women are more likely to cross the stability boundary and experience tissue-scale events driven by repolarization instability. Furthermore, analyses restricted to drugs whose mean QTc effects fall below the regulatory threshold indicate that the platform can mechanistically link multichannel drug block with sex hormone states, enhancing the mechanistic interpretability of drug risk assessment.

To further elucidate electrophysiological sources of sex differences in risk, we performed statistical and sensitivity analyses of risk drivers under multichannel block. Both sexes were sensitive to perturbations in *I*_Kr_, *I*_Ca,L_, and *I*_Ks_, but sex differences were mainly concentrated in *I*_Kr_, *I*_Ca,L_, *I*_Ks_, and *I*_Na_. This is consistent with prior simulations and clinical studies^15,28,34,60,61^ suggesting that women are more sensitive to disturbances of key repolarizing currents. To disentangle the relative roles of hormone states and ion-channel parameter backgrounds, we additionally conducted hormone-related counterfactual analyses by constructing four populations (normal male, normal female, androgen-deprived male, and androgenized female) and evaluating 2 × 2 counterfactual combinations. We observed that the acute hormonal modulation of ion channel parameters exhibited pronounced dependence on baseline parameter values, with a statistically significant non-additive interaction. Specifically, the effect of hormonal switching on arrhythmic risk varied substantially across distinct parameter backgrounds. Due to the threshold and non-linear characteristics of arrhythmia events themselves, and the fact that the risk endpoint in this study was a composite index of PVC, TWA, and RF, the above non-additive interaction would lead to the contribution decomposition based on the additive assumption (such as Shapley effects) being highly sensitive to the model setting and counterfactual combinations. Therefore, we placed the quantitative attribution results in the supplementary materials as a mechanism hint and sensitivity analysis, rather than treating it as an ‘explanation ratio’ that can be directly extrapolated to the clinical setting. Nevertheless, within the parameter range of the model setting in this study, the counterfactual analysis consistently indicated that sex hormones were an important contributor to the risk difference between men and women, even far stronger than the differences in ion channels themselves due to sex.

Finally, we used the virtual populations to predict time-resolved clinical ECG changes after dosing. Although the simulation results did not correspond exactly to the clinical median values at all time points, the model was able to reproduce the main dynamic forms of QTc changes and the overall concentration–effect trend under multiple drug and sex conditions without post-hoc fitting, suggesting that the constructed virtual population can capture the dominant mechanism of drug action on myocardial repolarization changes. For ranolazine and verapamil, the clinically observed changes in J-T_peak_c and T_peak_–T_end_ were small and variable, and the corresponding subject-level simulated slopes were concentrated close to zero (Supplementary material online, *Figure S6*). We therefore interpret the residual mismatch in these decomposed biomarkers cautiously. In this weak-signal regime, uncertainty in IC_50_/Hill estimates, possible pharmacologic effects not represented in the current parameterization, and the use of plasma concentration with a fixed free fraction as the exposure driver may materially influence the fitted slopes. Future extensions that incorporate myocardial exposure mapping, drug-binding kinetics, and harmonized ECG computation pipelines may improve quantitative agreement for decomposed biomarkers.

We emphasize that we used a 1D cable model and pseudo-ECG as a minimal tissue-scale implementation, enabling efficient evaluation of large virtual populations and capture of key instability events while preserving mechanistic interpretability. Incorporating 3D anatomy and more realistic body-surface projections may further improve the fidelity of T-wave morphology and derived metrics, thereby increasing clinical agreement at the waveform level.^62^ Additionally, in our sex modelling, we classified the differences into two categories: the gene expression level and the hormone status level. The gene expression level was constructed based on the differences in ion channel protein expression in the ventricular tissues of both sexes, thus to some extent, it incorporated the comprehensive results of long-term endocrine environments on expression. On this basis, we adopted an acute effect modular modelling strategy for the influence of sex hormones,^35,63,64^ which facilitated calibration with experimental ratios and provided clear mechanistic explanations. A limitation is that the current acute hormone module does not yet capture broader regulation mediated by signalling pathways, such as channel phosphorylation, calcium handling, and autonomic coupling.^37,65–68^

Despite these avenues for extension, the virtual population and mechanistic framework presented here can already support a range of translational applications, including sex-stratified risk assessment, decision-making in drug development, and mechanistic interpretation. Moreover, data-driven approaches could further complement this platform to accelerate candidate screening. For instance, deep generative models trained on the simulation data produced here could learn to directly sample physiologically plausible parameter sets conditioned on target clinical attributes (e.g. sex or QTc baseline), bypassing the current sampling-and-filtering loop and enabling on-demand construction of tailored virtual subpopulations. Furthermore, deep learning models capable of predicting ion-channel IC_50_ values directly from molecular structure could be coupled with the present framework, enabling cardiac safety evaluation at the drug design stage without prior electrophysiological experiments. These extensions would allow the mechanistic platform to be deployed earlier and more broadly in the drug development pipeline.

## Supplementary Material

euag133_Supplementary_Data

## Data Availability

Simulation data can be regenerated by rerunning the models described in Methods with the reported parameterization. Clinical ECG and concentration datasets used for external validation are publicly available from the cited sources. Summary data for the main figures and analyses are available from the corresponding author upon reasonable request.

## Code availability

Simulation and analysis code (CUDA-accelerated) will be made available to editors and reviewers during peer review and to readers upon reasonable request from the corresponding author. Code was developed and tested on an NVIDIA GeForce RTX 3090 GPU.
