# Supplementary material for: Sex-specific virtual population for the prediction and assessment of arrhythmia risk
Source: Europace. 2026 May 29;28(6):euag133. doi: 10.1093/europace/euag133 (PMC13317967; doi:10.1093/europace/euag133)
Supplement: euag133_Supplementary_Data [file euag133_supplementary_data.docx]

**Supplemental Information**

**Supplemental Methods**

**Filtering criteria**

**Single cell filtering:** Single-cell filters were applied to membrane voltage and intracellular calcium transients from representative Endo, M, and Epi cells. During baseline screening, membrane voltage and [Ca^2+^]_i_ were evaluated over one representative beat at PCL = 1 s. For membrane voltage, an implementation-level peak-detection rule was used to exclude abnormal paced waveforms: the trace had to contain exactly two detected peaks within one cycle, and the interval between these two peaks had to be ≤ 400 ms. In addition, resting membrane voltage measured at 900 ms had to lie between -96 and -76 mV. For intracellular calcium, [Ca^2+^]_i_ was required to exhibit one peak per cycle. Acceptable peak [Ca^2+^]_i_ ranges were 0.2-1 μM for Endo cells, 0.4-1 μM for M cells, and 0.2-1 μM for Epi cells.

**Cable pseudo-ECG filtering:** At the cable level, baseline filtering was based on pseudo-ECG morphology. QRS duration was required to fall between 60 and 100 ms. An auxiliary RS-interval filter, defined as $t_{S}-t_{R}$ (where $t_{R}$ and $t_{S}$ are the times of the R and S extrema, respectively), was required to be ≤ 100 ms to remove outlier activation patterns. QT interval was computed for each trace, but no fixed QT threshold was imposed at this stage because later population calibration was performed against clinical QTc distributions. Instead, the pseudo-ECG was required to show a normal single-T-wave morphology. Operationally, the trace was expected to contain two dominant positive peaks corresponding to R and T. Any additional positive peaks were allowed only if their amplitudes were < 0.02 mV. Likewise, no more than one valley was expected; if multiple valleys were present, their amplitudes were required to remain > -0.05 mV. These auxiliary peak/valley thresholds were model-specific signal-processing criteria used to exclude noisy or abnormally shaped pseudo-ECG traces.

**Drug information updates**

Sotalol: In our previous work,^1^ the human ether-à-go-go-related gene (hERG) IC_50_ of sotalol was set to 320 µM, which is close to values reported from automated patch-clamp assays. However, Orvos et al.^2^ reported a substantially higher potency based on manual patch-clamp measurements (IC_50_ ≈ 52 µM). In addition, Crumb et al.^3^ reported that sotalol inhibits Ca_V_1.2 only at millimolar concentrations (IC_50_ ≈ 7100 µM, Hill coefficient h ≈ 0.86). Accordingly, in the present study we updated the sotalol parameters by revising the hERG IC_50_ and the Ca_V_1.2 IC_50_ to better align with Comprehensive in vitro Proarrhythmia Assay (CiPA)-based measurements.

Dofetilide: The reported IC_50_ values for hERG inhibition vary considerably across different studies, ranging from 1 to 13 nM^3,4^. In this context, we adopt the value reported by Li et al.^5^, which is 4.9 nM (h = 0.9), as a reasonable estimate.

**2×2 counterfactual attribution analysis**

To disentangle the relative roles of sex-specific ion-channel parameter backgrounds and acute hormone state, we performed a 2×2 counterfactual attribution analysis. We constructed four virtual populations by crossing two binary factors: (i) baseline parameter background (male and female parameter sets used to generate the sex-specific populations) and (ii) androgen state (male-level testosterone and female-level testosterone). This yielded the following four counterfactual populations: normal male (male background and male hormone state), androgen-deprived male (male background and testosterone removed; serum testosterone set to 0 nM), androgenized female (female background and male-level testosterone; serum testosterone set to 35 nM), and normal female (female background and female hormone state). For each experimental condition (e.g., sympathetic stress and multichannel drug block), arrhythmic risk was quantified as the incidence of any tissue-scale event (PVC, TWA, or RF).

Let y_MM_, y_MF_, y_FM_, y_FF_ and denote transformed risks (log(1+risk)) in normal male, androgen-deprived male, androgenized female, and normal female populations, respectively. The overall sex difference was defined as $\Delta= y_{FF} - y_{MM}$. Contributions of ion-channel parameter background and hormone state were computed using a two-factor Shapley-style path average:

$$\begin{aligned} C_{G}=0.5\left[ \left( y_{FM}-y_{MM} \right)+\left( y_{FF}-y_{MF} \right) \right] \#\left( 10 \right) \end{aligned}$$

$$\begin{aligned} C_{H}=0.5\left[ \left( y_{MF}-y_{MM} \right)+\left( y_{FF}-y_{FM} \right) \right]\#\left( 11 \right) \end{aligned}$$

so that Δ = C_G_ + C_H_. Non-additive interaction between hormone switching and parameter background was quantified by the difference-in-differences term:

$$\begin{aligned} I = y_{FF}-y_{FM}-y_{MF}+y_{MM} \#\left( 12 \right) \end{aligned}$$

here, I ≠ 0 indicates that hormone switching effect depends on the ion channel background. We also report the four simple effects $H|G_{m}=y_{MF}-y_{MM}$, $H|G_{f}=y_{FF}-y_{FM}$, $G|H_{m}=y_{FM}-y_{MM}$, and $G|H_{f}=y_{FF}-y_{MF}$. Uncertainty summaries across drugs were obtained using a median and 95% CI over 109 drugs.

**Subject-level concentration-effect slope analysis**

To determine whether the simulated concentration-effect relationships in Fig. 8B reflected consistent behavior across virtual subjects or only population averages, we fitted a separate linear regression of biomarker change versus concentration for each virtual subject, drug, sex, and ECG biomarker using the same concentration-time points as in Fig. 8B. Histograms of these subject-specific slopes were then plotted. For reference, each panel also shows the slope obtained from the population-mean simulated responses (dashed line; corresponding to Fig. 8B) and the fitted clinical slope (solid line).

**Conduction analysis**

Apparent endocardium-to-epicardium conduction velocity was quantified retrospectively from the archived voltage traces in the final male and female virtual populations under baseline pacing (PCL = 1 s). In the archived 1D cable simulations, membrane voltage was saved at three fixed transmural sites only (cells 300, 153, and 6) and sampled at 1 ms intervals. Because a full spatial voltage field was not stored, local intralayer conduction velocity could not be reconstructed reliably. We therefore report the overall endocardium-to-epicardium apparent conduction velocity.

For each virtual subject, activation time at the endocardial and epicardial sampled sites was defined as the time of upward threshold crossing of membrane voltage ($V_{m}$ = -10 mV). To reduce discretization error from the 1 ms output sampling, activation time was estimated by linear interpolation between the two adjacent stored samples that bracketed the threshold:

$$t_{act}=t_{k}+\Delta t\times\frac{V_{th}-V_{k}}{V_{k+1}-V_{k}}$$

Where, $V_{th}$ is -10mV, $V_{k}<V_{th}\leq V_{k+1}$ and $\Delta t$ = 1ms. The conduction distance was taken as the separation between cells 300 and 6 along the transmural cable: $\Delta x$ = (300 - 6) × 0.004 cm = 1.176 cm. Apparent conduction velocity was then calculated as:

$$CV= \frac{\Delta x}{t_{act,epi}-t_{act,endo}}$$

**Alternative sympathetic-drive sensitivity analysis in LQTS cohorts**

In an additional sensitivity analysis, sympathetic drive in the LQTS simulations was represented by concurrent enhancement of I_Ca,L_ and I_Ks_, rather than enhancement of I_Ca,L_ alone. Because the admissibility filter in the main pipeline depends on the stress protocol(ISO effect), we re-screened the original male and female model population(Fig.1B) under 2.0×I_Ca,L_ + 2.0×I_Ks_ and then generated LQT1-LQT3 cohorts using the same parameter ranges as in the main analysis. This additional screening condition is consistent with that of Stage 2, except that 2.0×I_Ca,L_ has been changed to 2.0×I_Ca,L_ + 2.0×I_Ks_. The resulting screened male and female candidate populations were then distribution-matched to the same clinical QTc targets as in the main text and used as the parent cohorts for generating the LQT1-LQT3 populations. QTc distributions were evaluated at baseline, 2.0×I_Ca,L_ + 1.0×I_Ks_, and 2.0×I_Ca,L_ + 2.0×I_Ks_. Under fixed 2.0×I_Ca,L_, I_Ks_ was further swept from 1.0× to 2.0× to quantify changes in absolute risk and mean ΔQTc. Sex effects were summarized as odds ratios (ORs) from subtype-specific logistic regression.

**Supplemental Table**

**Table S1.** The scaling coefficient range for the preset parameters of the male and female groups is based on multiple studies.^6–9^ The base values of the parameters are from Tomek et al.,^10^ where the base value of the dshift is 1 mV, and the true value of the parameter is equal to the scaling coefficient multiplied by the base value.

| Parameter | Male | | | Female | | |
| --- | --- | --- | --- | --- | --- | --- |
|  | Endo | M | Epi | Endo | M | Epi |
| G_Na_ | [0.5, 1.5] | 1 | 1 | [0.5, 1.5] | 1 | 1 |
| G_NaL_ | [0.1, 2] | [0.71, 1.11] | [0.32, 0.83] | [0.0877, 1.75] | [0.71, 1.11] | [0.31, 0.8] |
| G_to_ | [0.1, 2] | [2, 4.82] | [2, 4.82] | [0.1, 2] | [2, 4.82] | [2, 4.82] |
| P_Ca_ | [0.4, 1.05] | [1.25, 2.79] | [0.68, 1.78] | [0.64, 1.68] | [1.25, 2.79] | [0.76, 1.99] |
| G_Kr_ | [0.05, 1.2] | [0.81, 1.31] | [0.83, 2.11] | [0.04, 0.95] | [0.8, 1.32] | [0.84, 2.13] |
| G_Ks_ | [2.8, 22.75] | [0.8, 1.13] | [1.18, 1.52] | [2.24, 18.2] | [0.8, 1.32] | [1.18, 1.52] |
| G_K1_ | [0.1, 2] | [1.37, 2.23] | [0.89, 2.29] | [0.085, 1.7] | [1.37, 2.23] | [0.74, 1.91] |
| G_NCX_ | [0.1, 2] | [1.36, 1.97] | [0.88, 1.84] | [0.1, 2] | [1.36, 1.97] | [0.88, 1.84] |
| P_NaK_ | [0.1, 2] | [0.63, 0.85] | [0.8, 1.05] | [0.1, 2] | [0.63, 0.85] | [1.3, 2.1] |
| G_Kb_ | [0.1, 2] | 1 | [0.48, 0.63] | [0.054, 1.088] | 1 | [0.45, 0.59] |
| J_rel_ | 1 | [1.27, 2.03] | [0.8, 1.25] | 1 | [1.27, 2.04] | [0.62, 0.97] |
| J_up_ | 1 | [0.83, 1.23] | [0.68, 1.86] | 1 | [0.83, 1.23] | [0.82, 2.25] |
| d_shift_ | [0, 11] | 1 | 1 | [0, 11] | 1 | 1 |
| [Na]_i_ | [0.8, 1.4] | 1 | 1 | [0.8, 1.4] | 1 | 1 |
| [K]_o_ | [0.8, 1.2] | 1 | 1 | [0.8, 1.2] | 1 | 1 |

**Table S2.** Hormone concentrations were sampled within the physiological ranges listed here. Concentrations were expressed in nM and directly used in the Hill-type scaling functions above.

| Hormone | Male | Female |
| --- | --- | --- |
| Testosterone | 10.0 – 35.0 nM | 0.5 – 2 nM |
| Progesterone | 0 nM | 0.6 – 5 nM |
| Estradiol | 0 nM | 0.1 – 1 nM |

**Table S3**. Comparison of representative computational cardiac electrophysiology studies evaluating drug effects on cardiac repolarization, and their complementary relationship to the present work.

| **Study** | **Primary application** | **Scale** | **Sex-specific** | **Main outputs** | **Main strengths** | **Complementary relationship to present study** |
| --- | --- | --- | --- | --- | --- | --- |
| Passini et al., 2017^11^ | Drug safety / TdP prediction | Population of 1,213 human ventricular AP models (cell-scale) | No | AP biomarkers and repolarization abnormalities across 62 compounds | i) Landmark human in-silico drug trial;  ii) Benchmarked against rabbit wedge and hiPSC-CM data;  iii) Achieved 89% accuracy in clinical proarrhythmic risk prediction | i) Cell-scale only;  ii) No propagated tissue or pseudo-ECG behavior;  iii) No sex stratification |
| Fogli Iseppe et al., 2021^12^ | Sex-specific TdP classification | Male/female cardiac myocyte simulations with machine learning | Yes | Sex-specific TdP classifiers | i) Demonstrates that male and female TdP classifiers differ meaningfully;  ii) Directly supports sex-aware safety assessment | i) Restricted to cell-scale;  ii) No tissue-level instability endpoints or propagated ECG readouts |
| Hwang et al., 2019^13^ | 3D ECG-based drug safety assessment | 3D ventricles and torso body-surface ECG simulation | No | QT, JT_peak_, T_peak_-T_end_, arrhythmia behavior for 7 drugs | Explicit 3D body-surface ECG framework for CiPA-related biomarkers under multichannel block | i) Limited to 7 drugs;  ii) Did not capture the clinically observed non-increasing JT_peak_ behavior for verapamil and ranolazine |
| Peirlinck et al., 2021^9^ | Sex differences in drug-induced arrhythmogenesis | Multiscale whole-heart modeling with machine learning | Yes | Critical drug concentration / sex-specific classifier | i) Integrates ion-channel activity, tissue conductivity, and cardiac geometry;  ii) Reported an approximately 7-fold lower critical dofetilide concentration in women | Focused on sex-specific susceptibility thresholds rather than large-scale multi-drug virtual trials with clinical concentration-ECG validation |
| Dominguez-Gomez et al., 2024^14^ | Fast sex-specific QT prediction for drug safety | 3D biventricular simulator and sex-specific emulators | Yes | Arrhythmia classifier and ΔQT emulator | i) High-fidelity sex-specific 3D framework;  ii) Emulators trained on 900 simulations with approximately 4% average error;  iii) Enables rapid multi-dose testing | i) Primarily QT-based; does not account for phenotype or anatomical variability; ii) Emulators are designed to complement rather than replace full simulations |
| Dasí et al., 2024^15^ | Efficacy-oriented treatment stratification | 800 virtual AF patients with variability in anatomy, electrophysiology, and tissue structure | No | Response to 12 secondary treatments after AF recurrence | i) Illustrates translational value of large virtual-patient cohorts;  ii) Validated across scales from ionic currents to ECG | Atrial AF efficacy study, not directly applicable to ventricular drug-induced repolarization safety |
| Present study | Sex-specific ventricular pro-arrhythmic risk under LQTS, sympathetic stress, and 109 drugs | 1D ventricular cable with 30,000 virtual subjects per sex | Yes | QTc/J-T_peak_c/T_peak_-T_end_, PVC/TWA/RF, sex-specific risk ratios | Scalable sex-aware tissue-scale repolarization-risk analysis with direct clinical concentration-ECG comparison | 1D cable model cannot capture re-entrant arrhythmias and offers lower anatomical and waveform fidelity than full 3D frameworks |

**Supplemental Results and Figures**


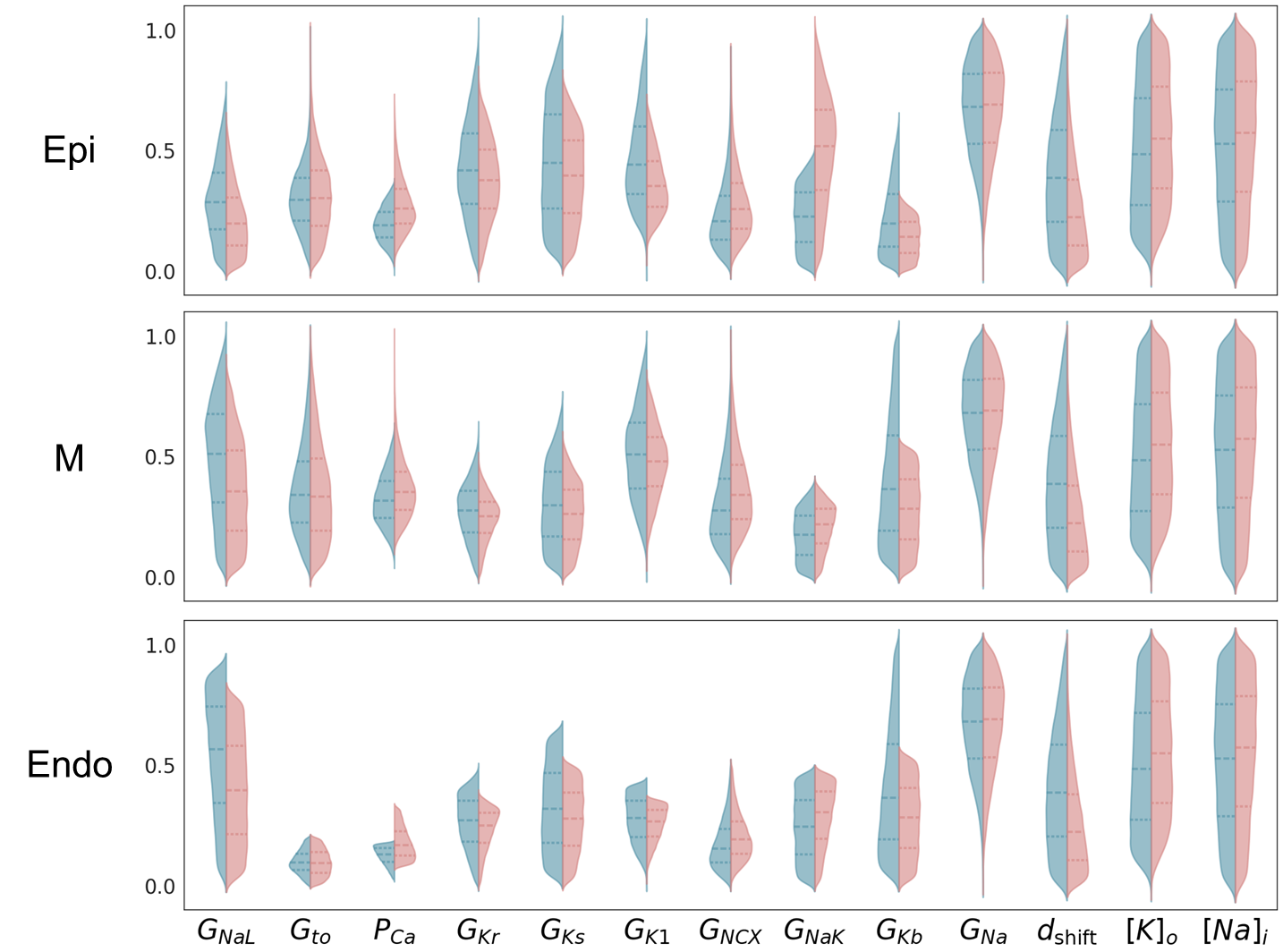


**Fig. S1. Parameter distributions of the normal male and female population.** The figure shows violin plots of the parameter distributions of the three layers of the endocardium (bottom), myocardium middle layer (middle), and epicardium (top). The left half of the violin plots represents the parameters of males, and the right half represents those of females. The range of each parameter has been normalized to between 0 and 1. In each violin plot, the middle dashed line represents the median, while the lower and upper dashed lines represent the first and third quartiles, respectively.


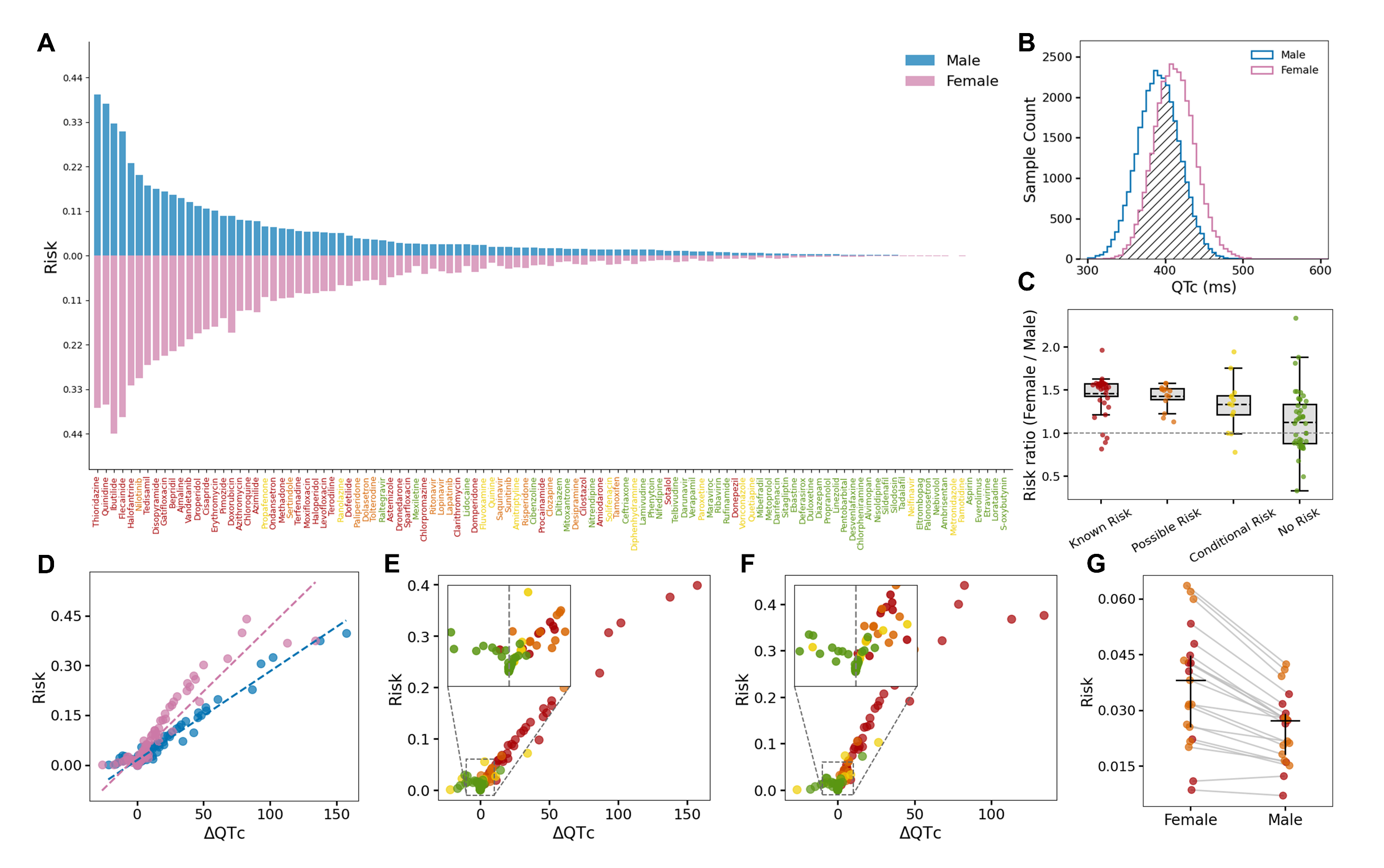


**Fig. S2. Drug risk assessment results for normal male and female groups with consistent QTc distribution.** **(A)** Arrhythmic event risk for 109 drugs in the specific (same QTc distribution) male and female virtual populations. Drugs are ordered by male risk from high to low. **(B)** The overlapping area of the two groups was used as the subpopulation for evaluation, and their QTc distributions were almost identical. The number of male population is 22,379, $\bar{QTc}\pm\sigma$= 401.74 ± 23.06, while female population number is same, $\bar{QTc}\pm\sigma$ = 402.26 ± 24.00. **(C)** Female-to-male risk ratio aggregated by clinical risk category, and its association with risk category (Spearman ρ = -0.549, p = 6.031×10^-10^). **(D)** Relationship between drug-induced ΔQTc and event risk across the 109 drugs, with linear fits. Regression parameters in females: k = 0.00391, b = 0.0263 (R^2^ = 0.861); in males: k = 0.00268, b = 0.0146 (R^2^ = 0.950). **(E)** and **(F)** are scatter plots of ΔQTc and event risk induced by drugs in male and female population, respectively. The subplots show the range of ΔQTc from -10 to 10 ms. The colors of the scatter points are consistent with the labels of the drugs. **(G)** Sex differences in risk for drugs labeled as Known Risk and Possible Risk. Horizontal black lines denote medians, and vertical black lines show interquartile ranges. Paired Wilcoxon test, p < 0.001.


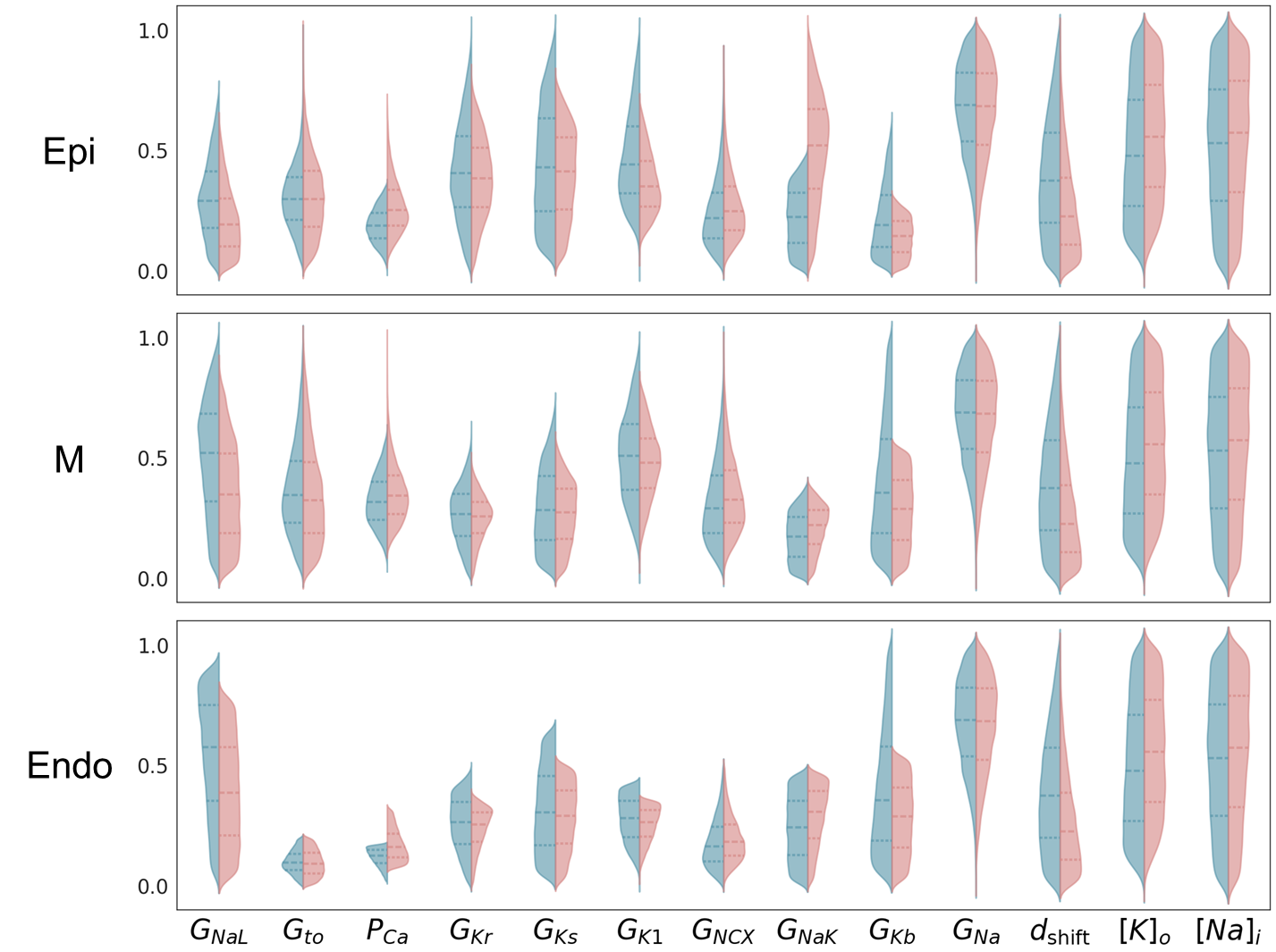


**Fig. S3. Parameter distributions of the normal males and females with same QTc distribution.** The figure shows violin plots of the parameter distributions of the three layers: endocardium (bottom), myocardium middle layer (middle), and epicardium (top). The left half of the violin plots represents the parameters of males, and the right half represents those of females. The range of each parameter has been normalized to between 0 and 1. In each violin plot, the middle dashed line represents the median, while the lower and upper dashed lines represent the first and third quartiles, respectively.

In the 2×2 counterfactual design, we mainly perturbed androgen (androgenization and androgen deprivation states) to quantify how the acute regulation related to testosterone interacts with the background of ion channel parameters. Estradiol and progesterone were included to define the baseline female cohort (limited to the follicular phase or early luteal phase to avoid the approximately 10-fold progesterone surge in the mid-luteal phase), but were not independently perturbed to avoid introducing menstrual cycle heterogeneity and a difficult-to-identify high-dimensional counterfactual space.


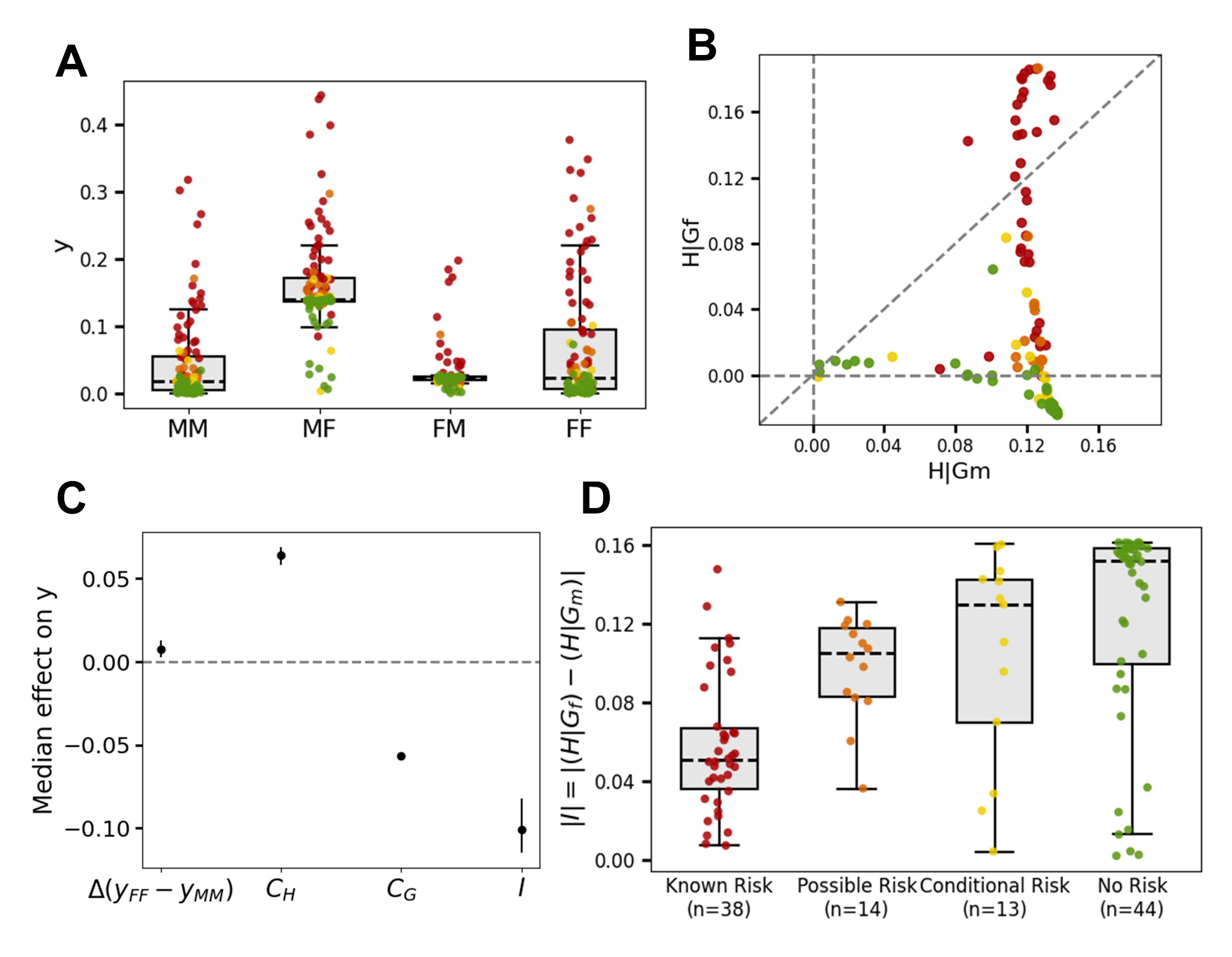


**Fig. S4. Hormone-by-background 2×2 counterfactual analysis under multichannel drug block.** **(A)** Distribution of y across the four groups: MM (male ion channels with male hormones), MF (male ion channels with female hormones), FM (female ion channels with male hormones), and FF (female ion channels with female hormones). The point color means drug label, red is Known Risk, orange is Possible Risk, yellow is Conditional Risk, and green is No Risk. **(B)** Background-dependence of hormone switching effects across drugs, comparing $H|Gm$ versus $H|Gf$. The diagonal dash line is y = x, which indicates no effect modification. **(C)** Summary of overall contrast $\Delta= y_{FF} - y_{MM}$, Shapley-style average contributions C_H_ and C_G_, and non-additive interaction term I. Points show bootstrap medians across drugs and error bars indicate bootstrap 95% CI. **(D)** Distribution of $|I|$ stratified by drug categories. Smaller $|I|$ indicates weaker effect modification of hormone switching by ion channel parameter background.


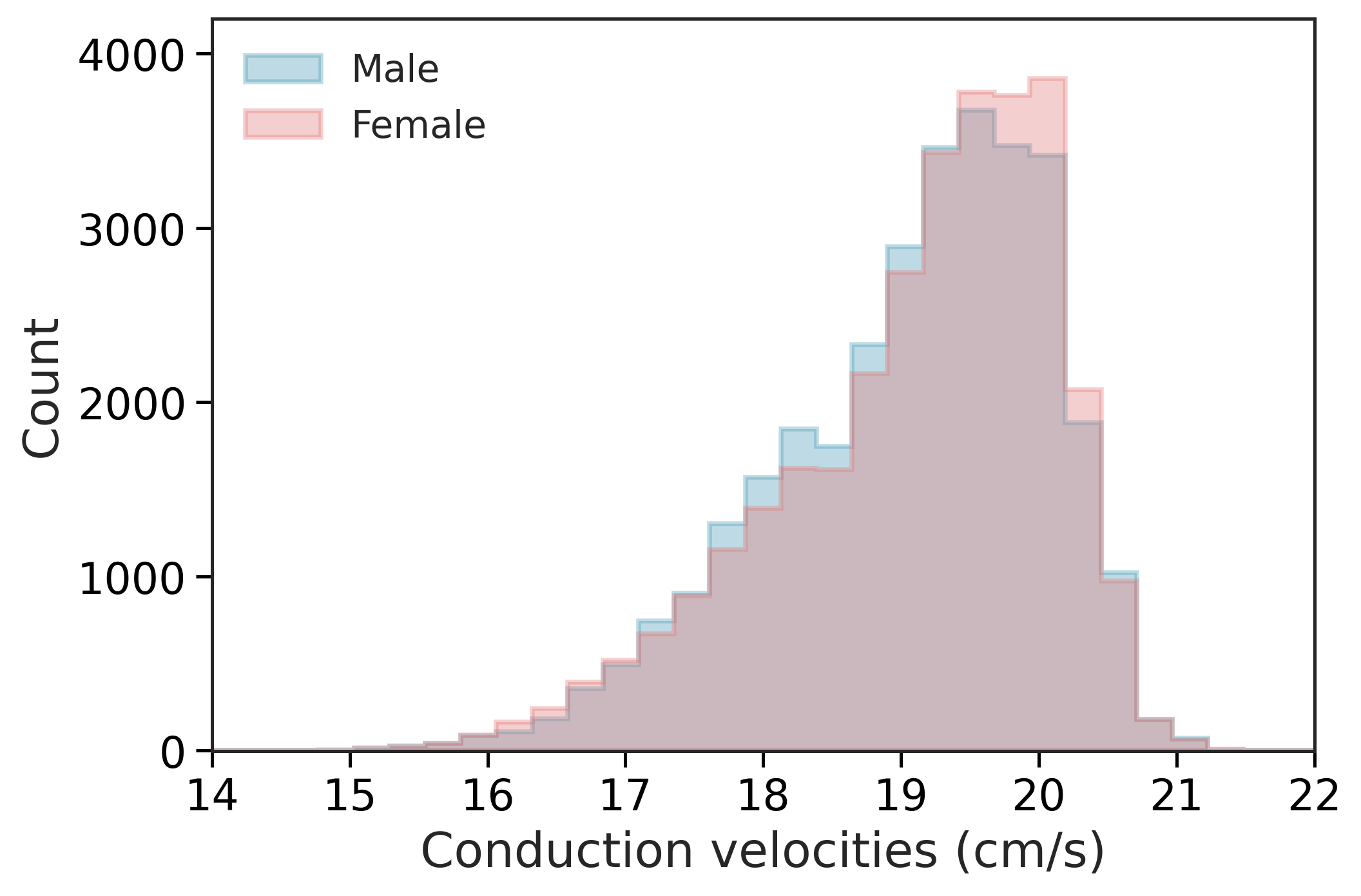


**Fig. S5. Distributions of conduction velocity in the male and female virtual populations.**

Apparent conduction velocity was computed retrospectively from the distance between the sampled endocardial and epicardial sites (cells 300 and 6) and the corresponding activation delay, with activation times defined by upward threshold crossing of membrane voltage and linear interpolation between consecutive 1 ms samples. Male and female distributions were highly similar, consistent with the identical diffusion coefficient and the absence of sex-specific cell-to-cell coupling differences in the present model.


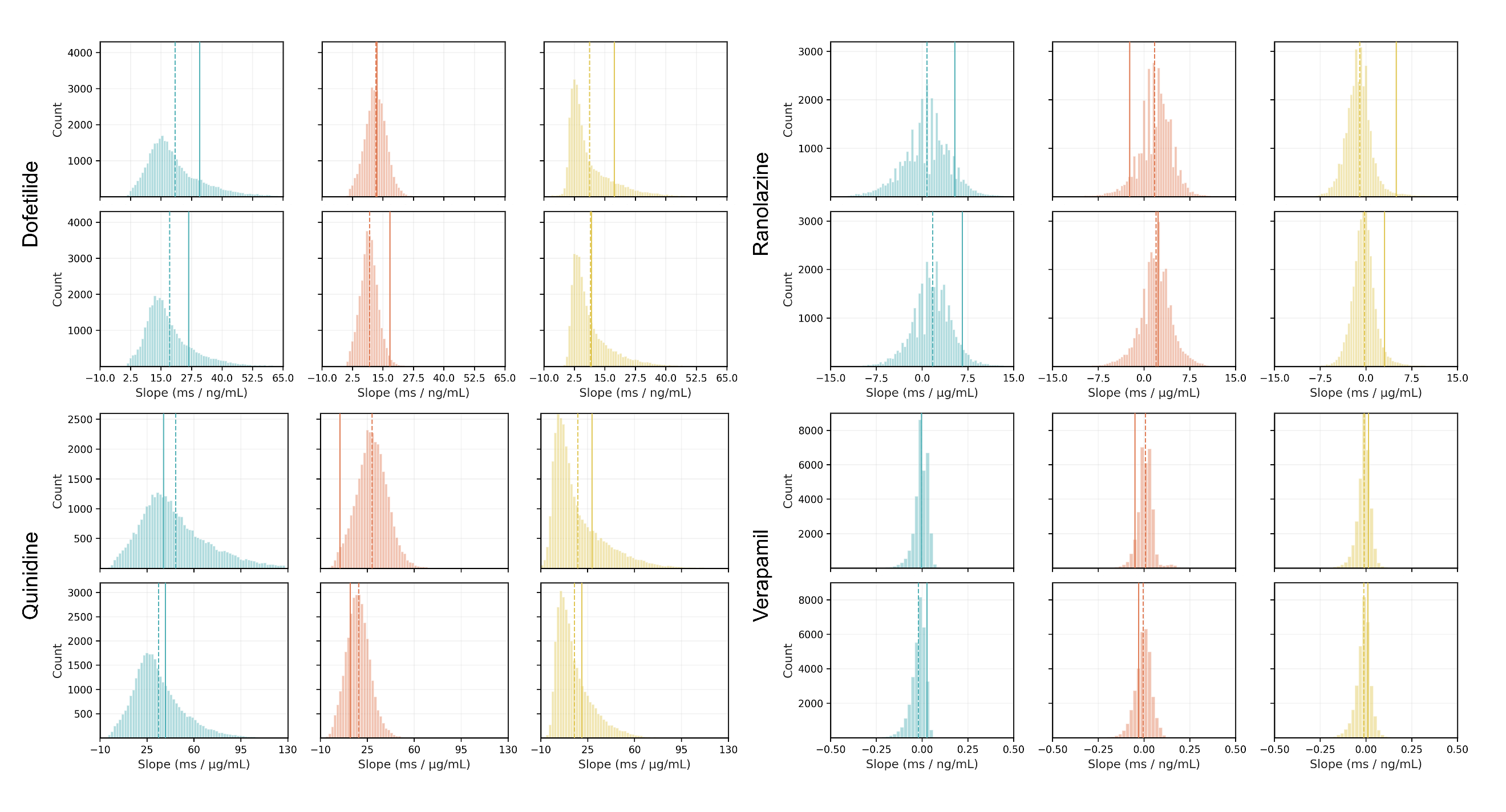


**Fig. S6. Distributions of subject-specific concentration-effect slopes in the virtual populations.** For each drug, ECG biomarker, and sex-specific virtual population, a linear concentration-effect slope was fitted separately for each virtual subject using the same concentration-time points as in Fig. 8B. Histograms show the distributions of these subject-specific slopes. Columns correspond to QTc, J-T_peak_c, and T_peak_-T_end_, respectively. Within each drug, the upper and lower rows represent virtual male and female populations. Dashed vertical lines denote the slopes obtained by fitting the population-mean simulated responses shown in Fig. 8B, whereas solid vertical lines denote the fitted clinical slopes.


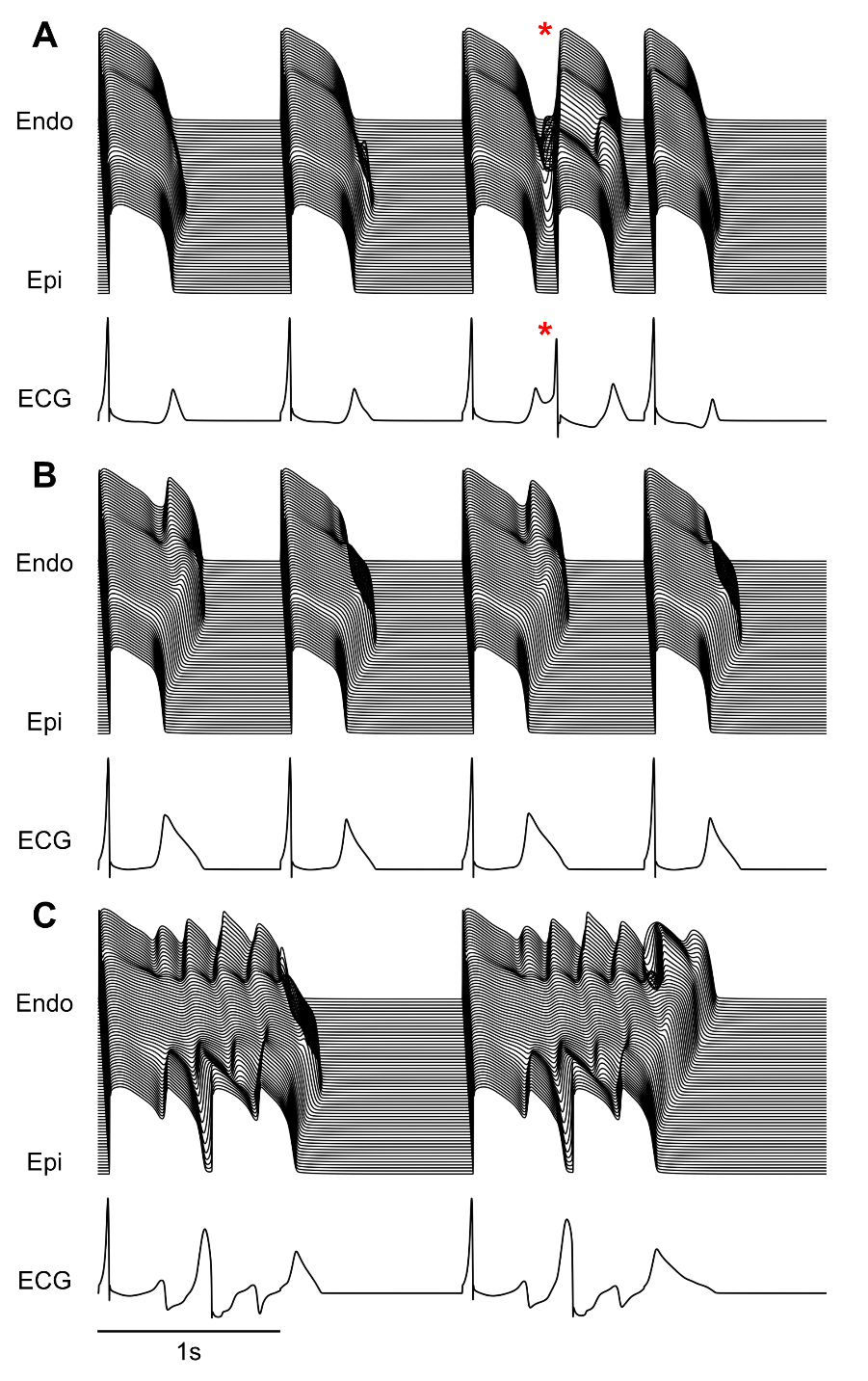


**Fig. S7. Representative examples of PVCs, TWA, and RF in 1D cable simulations.** In each panel, the upper plot shows the space-time evolution of membrane voltage along the Endo-M-Epi cable, and the lower trace shows the corresponding pseudo-ECG. (A) Example of premature ventricular complexes (PVCs), marked by red asterisks, illustrating premature ectopic depolarizations before the next paced beat. (B) Example of T-wave alternans (TWA), showing beat-to-beat alternation in T-wave morphology, marked by an L-S-L-S pattern. (C) Example of 2:1 repolarization failure (RF), in which every other paced beat fails to repolarize normally before the next stimulus.

**Structural sensitivity of sex-specific LQTS predictions to altered midmyocardial-region width**

To demonstrate the robustness of our method, we tested whether the sex-specific LQTS conclusions depended on the assumed width of the M region in the 1D cable. Under each altered M-region configuration, the full pipeline described in the main text Methods (parameter sampling, multi-stage filtering, QTc-matched calibration, and LQTS construction) was re-executed from scratch, with all sampling ranges, filtering criteria, and calibration targets unchanged, only the transmural extent of the M region was altered. Reducing the M-region width by 50% while preserving total cable length produced modest shifts in the retained QTc distributions, but the sex-specific pattern was preserved. Under the reduced-M configuration, the retained normal cohorts remained well separated in QTc (391.62 ± 27.42 ms in males, and 412.13 ± 25.87 ms in females), and the derived female LQT1-LQT3 cohorts continued to exhibit longer QTc than the corresponding male cohorts. Most importantly, female arrhythmic risk remained higher than male risk across all subtypes, with in-silico ORs of 2.94, 2.86, 2.29, and 2.74 for LQT1, LQT2, LQT3, and pooled LQTS, respectively. The same qualitative conclusion was observed when the M-region width was increased by 50%. These findings indicate that the principal sex-specific risk predictions are structurally robust and do not depend on one specific choice of M-region extent.


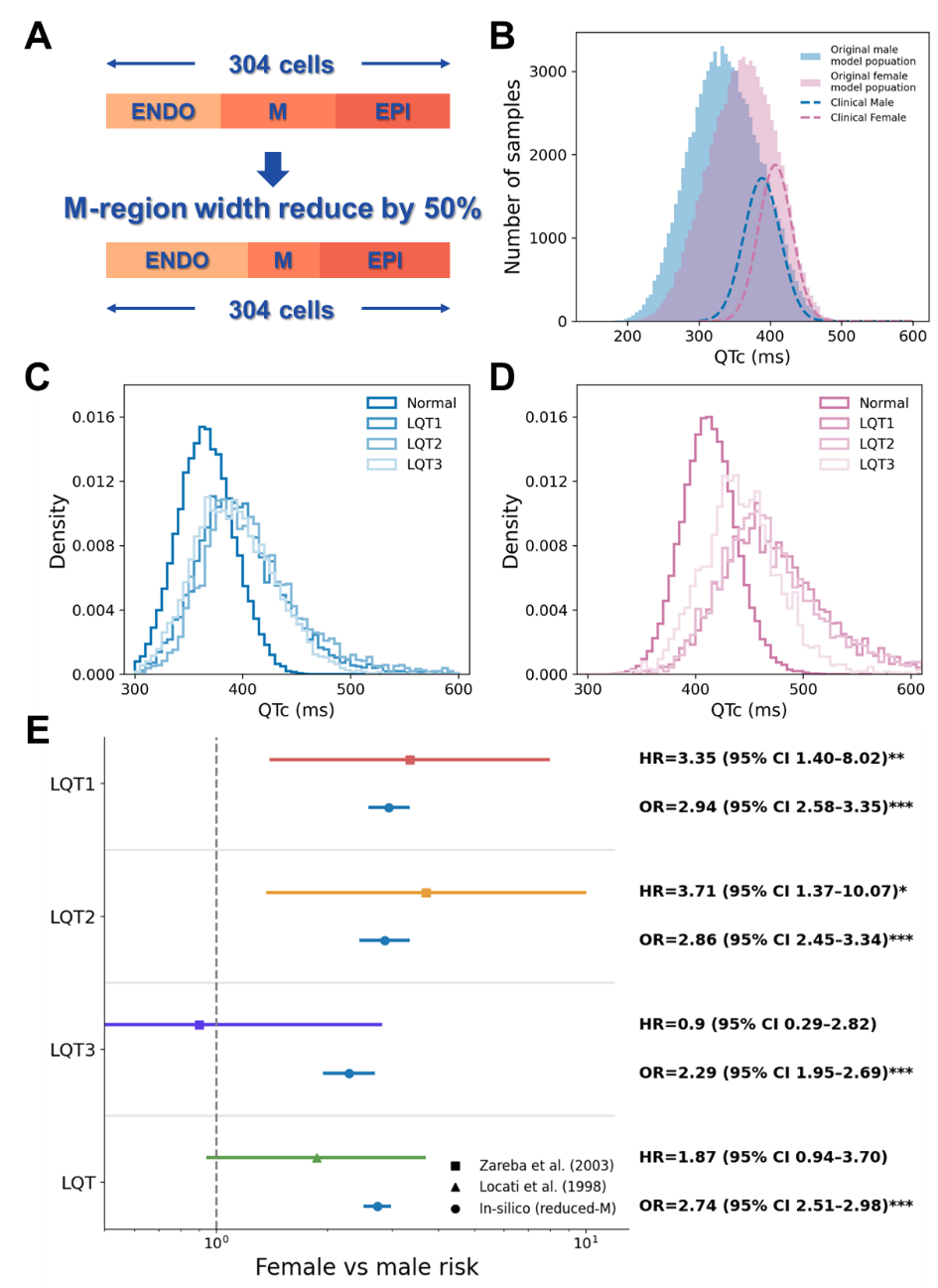


**Fig. S8. Structural sensitivity of sex-specific LQTS predictions to a 50% reduction in midmyocardial-region width.** (A) Schematic of the modified 1D cable geometry. The total cable length was kept constant at 304 cells, while the midmyocardial region width was reduced by 50% and the Endo and Epi regions were increased proportionally. (B) QTc distributions of the original male and female candidate populations generated under the reduced-M geometry, together with the corresponding clinical reference distributions. (C-D) Baseline QTc distributions of the male and female LQT1-LQT3 cohorts derived from the retained reduced-M normal cohorts. (E) Female-to-male effect sizes for LQT1, LQT2, LQT3, and pooled LQTS under the reduced-M configuration, shown together with published clinical estimates.


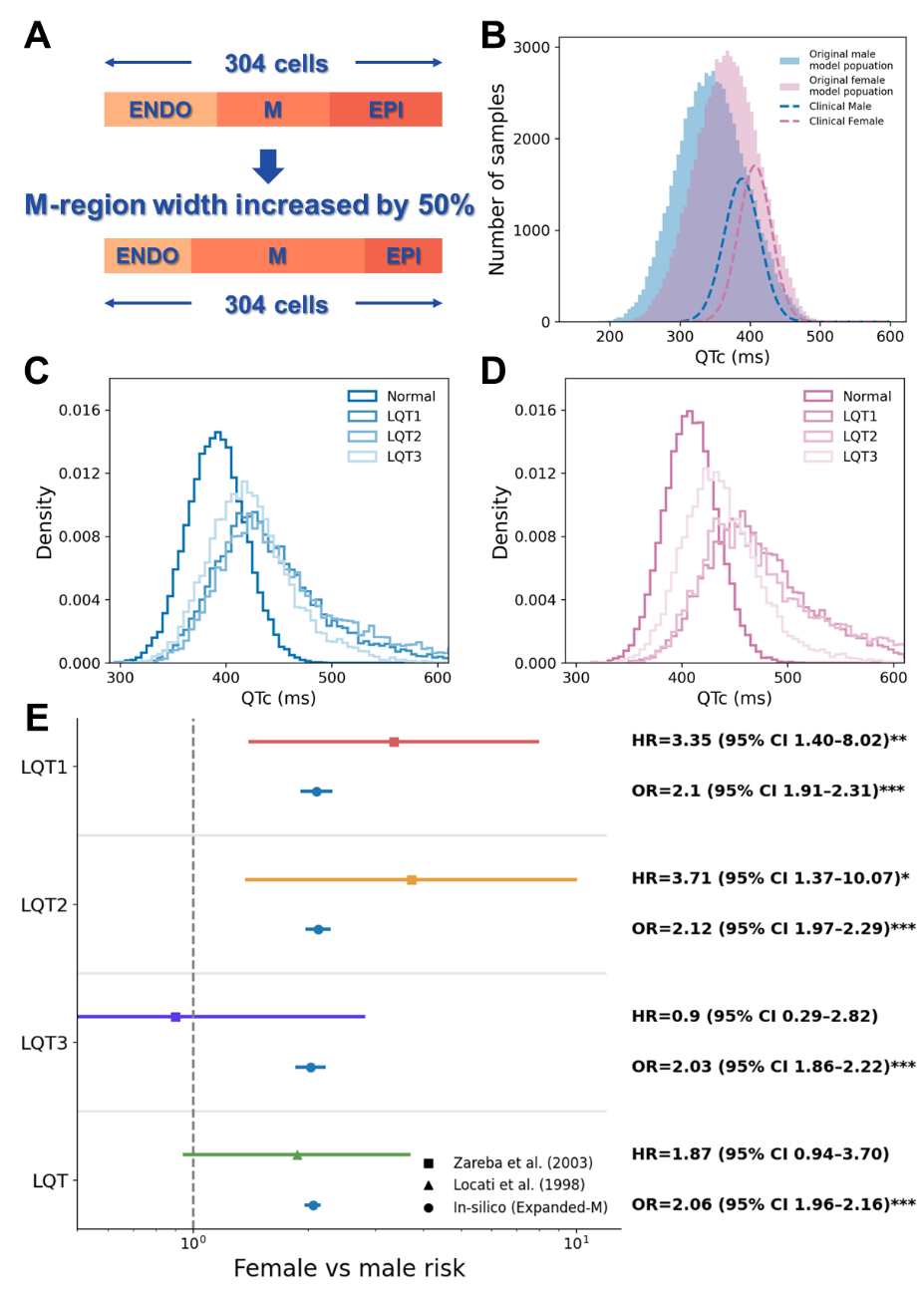


**Fig. S9. Structural sensitivity of sex-specific LQTS predictions to a 50% increase in midmyocardial-region width.** (A) Schematic of the modified 1D cable geometry. The total cable length was kept constant at 304 cells, while the midmyocardial region width was increased by 50% and the Endo and Epi regions were adjusted accordingly. (B) QTc distributions of the original male and female candidate populations generated under the expanded-M geometry, together with the corresponding clinical reference distributions. (C-D) Baseline QTc distributions of the male and female LQT1-LQT3 cohorts derived from the retained expanded-M normal cohorts. (E) Female-to-male effect sizes for LQT1, LQT2, LQT3, and pooled LQTS under the expanded-M configuration, shown together with published clinical estimates.

**Alternative sympathetic-drive sensitivity analysis in LQTS cohorts**

To ensure consistency between the stress protocol used during virtual population generation and the one subsequently applied to induce arrhythmias, we re-screened the original populations according to the updated filtering criteria (see Supplemental Methods). Baseline QTc distributions of the re-screened cohorts remained very similar to those obtained under the original pipeline(Fig. 3). Under 2.0×I_Ca,L_ alone, QTc distributions shifted rightward in all subtypes, whereas concurrent I_Ks_ enhancement partially reversed this prolongation and reduced absolute arrhythmic risk. This rescue was heterogeneous rather than a simple uniform leftward translation of the entire cohort, producing broader post-stimulation QTc distributions in some subtype-sex combinations. Importantly, however, female-to-male in-silico ORs remained above 1 under both 2.0×I_Ca,L_ + 1.0×I_Ks_ and 2.0×I_Ca,L_ + 2.0×I_Ks_ across LQT1, LQT2, LQT3, and pooled LQTS, indicating that the principal conclusion of higher female susceptibility was preserved under the revised sympathetic-drive formulation.

The effect of I_Ks_ enhancement was subtype dependent. Risk attenuation was most pronounced in LQT1, consistent with our formulation of LQT1 as partial IKs block rather than complete loss of channel function, leaving residual I_Ks_ that can still be augmented. In LQT2, concurrent I_Ks_ enhancement shortened QTc but produced a more limited reduction in absolute risk. This pattern is qualitatively consistent with recent rabbit work^16^ showing that I_Ks_ activation can shorten APD and QT in LQT2 without necessarily reducing whole-heart arrhythmia formation, while having no effect in a dominant-negative KCNQ1-Y315S LQT1 model with functionally absent I_Ks_. In LQT3, QTc also shortened as I_Ks_ was increased, but the reduction in absolute risk was modest, we therefore do not interpret sympathetic stimulation as strongly stabilizing in this subtype.


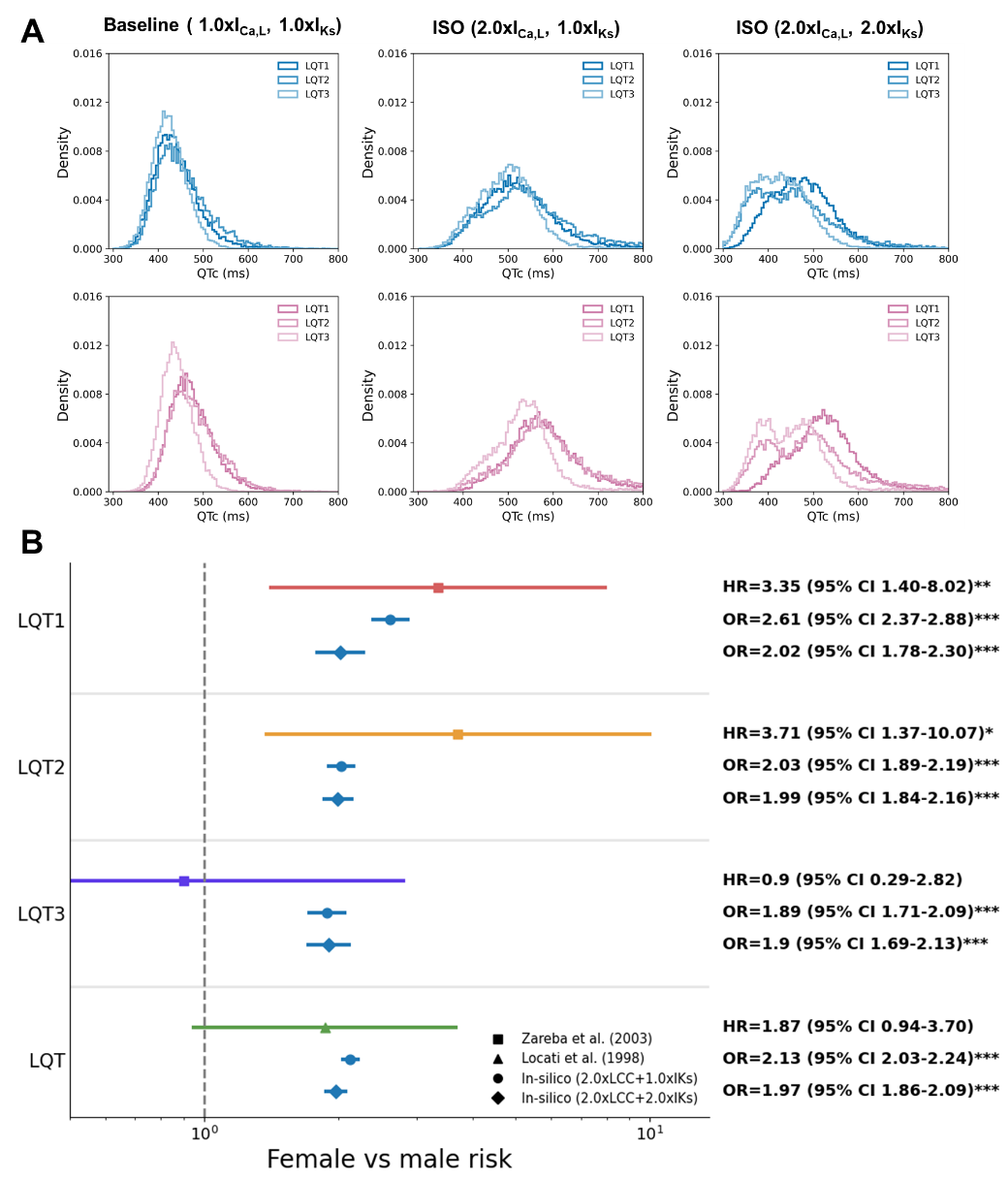


**Fig. S10. Sensitivity of sex-specific LQTS predictions to an alternative sympathetic-drive formulation with concurrent I_Ca,L_ and I_Ks_ enhancement.** (A) QTc distributions of the re-screened sex-specific LQTS cohorts. The top row shows males and the bottom row represents females. From left to right, panels correspond to baseline condition (1.0×I_Ca,L_, 1.0×I_Ks_), 2.0xI_Ca,L_ with 1.0xI_Ks_, and 2.0×I_Ca,L_ with 2.0×I_Ks_. Curves correspond to LQT1, LQT2, and LQT3. (B) Female-to-male risk ratios in LQTS under the alternative sympathetic-drive formulation. Squares and triangles denote clinical estimates from prior studies, whereas circles and diamonds denote in-silico odds ratios (ORs) for 2.0×I_Ca,L_ + 1.0×I_Ks_ and 2.0×I_Ca,L_ + 2.0×I_Ks_, respectively. The dashed vertical line marks an effect size of 1.


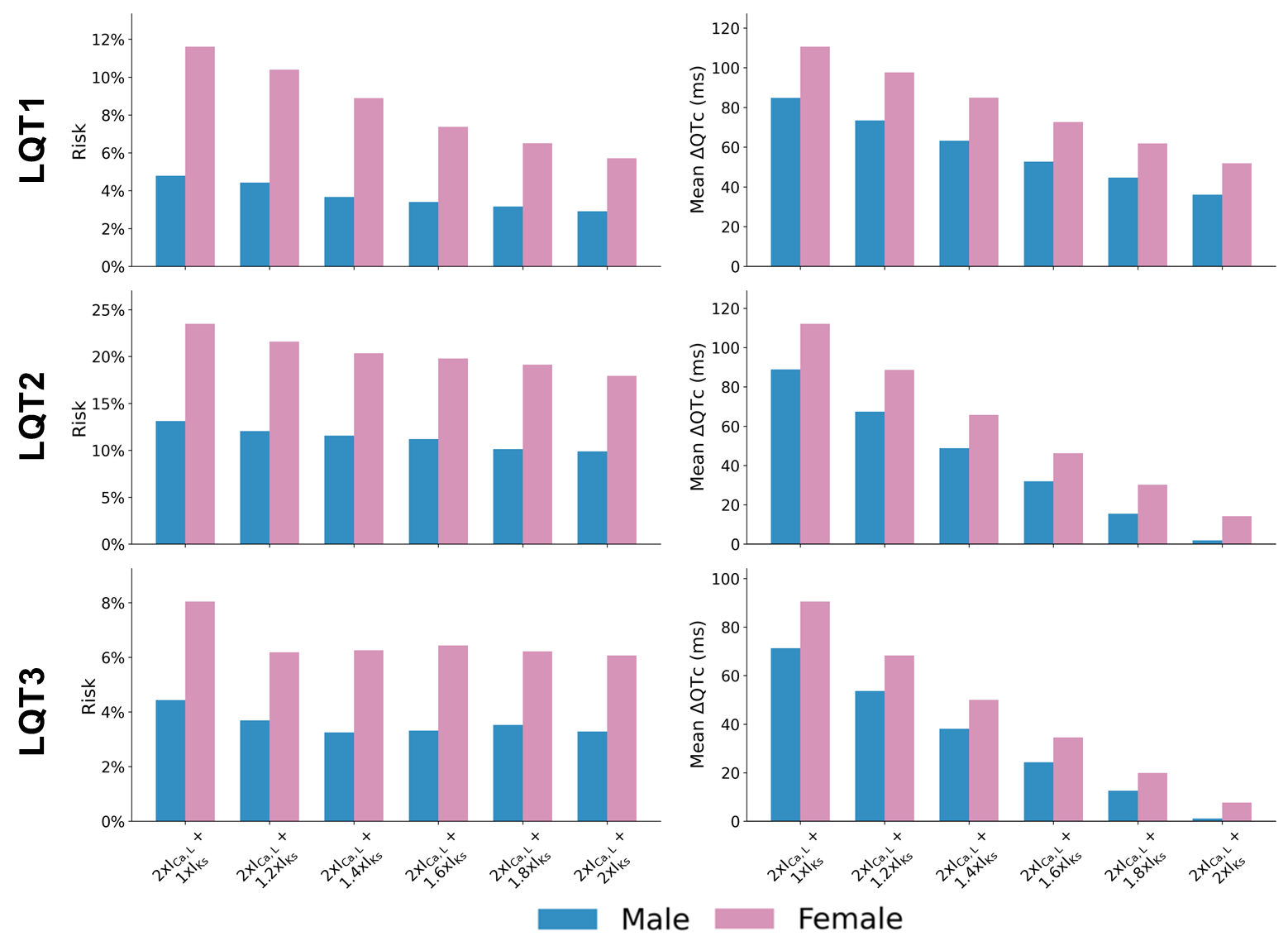


**Fig. S11. Effects of graded I_Ks_ enhancement on absolute arrhythmic risk and mean ΔQTc in re-screened sex-specific LQTS cohorts.** For each subtype, simulations were performed under fixed 2.0×I_Ca,L_ while I_Ks_ was progressively increased from 1.0× to 2.0×. Blue bars indicate males and pink bars indicate females. Left panels show the incidence of any arrhythmic event (PVC, TWA, or RF), and right panels show mean ΔQTc relative to the corresponding baseline LQTS condition without I_Ca,L_ and I_Ks_ scaling.

**Alternative sympathetic-drive sensitivity analysis in drug simulations**

To assess the robustness of the drug simulation results to the sympathetic-drive formulation, we repeated the 109-drug virtual trials using the re-screened normal male and female populations described above, with the proarrhythmic challenge defined as 2.0×I_Ca,L_ + 2.0×I_Ks_. Compared with the main analysis, concurrent IKs enhancement attenuated absolute drug-induced risk and reduced the separation between sexes. Consequently, the association between the female-to-male risk ratio and clinical risk category became less pronounced, and the risk-versus-ΔQTc regression slopes were shallower for both sexes. However, the female slope remained greater than the male slope. Thus, although the quantitative sex separation was attenuated under concurrent I_Ca,L_ and I_Ks_ enhancement, the qualitative conclusion that females exhibit higher drug-induced proarrhythmic susceptibility remained unchanged.


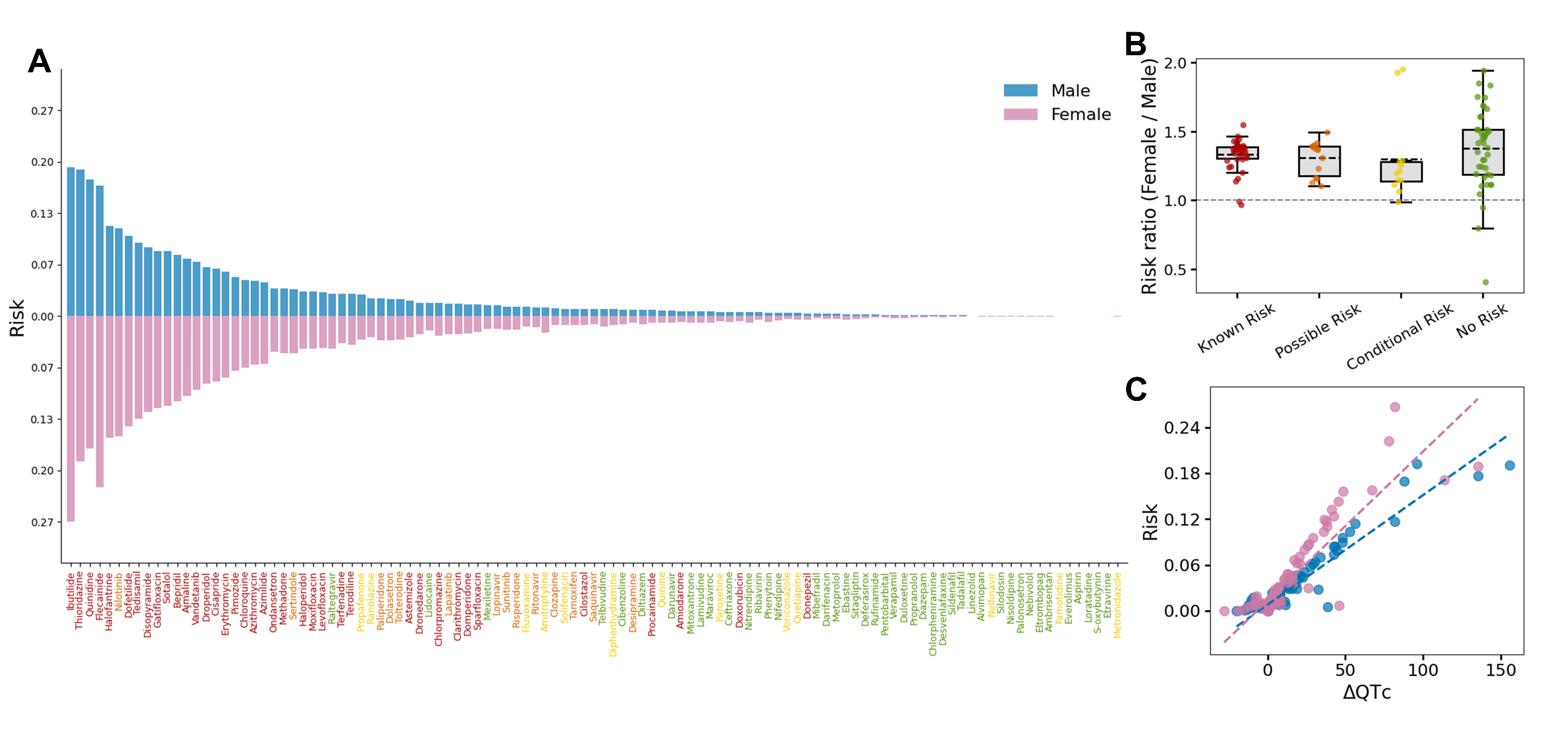


**Fig. S12. Sensitivity of drug-induced sex differences to an alternative sympathetic-drive formulation with concurrent I_Ca,L_ and I_Ks_ enhancement.** (A) Arrhythmic event risk for 109 drugs in the re-screened male and female virtual populations under 2.0×I_Ca,L_ + 2.0×I_Ks_. Drugs are ordered by male risk from high to low, and colors indicate clinical risk categories. (B) Female-to-male risk ratio aggregated by clinical risk category under the alternative sympathetic-drive formulation. The association with risk category was weak (Spearman ρ = 0.084, p = 0.392). (C) Relationship between drug-induced ΔQTc and event risk across the 109 drugs under the alternative sympathetic-drive formulation, with linear fits. Regression parameters were y = 0.00143x + 0.00792 (R^2^ = 0.901) in males and y = 0.00195x + 0.013 (R^2^ = 0.800) in females. Despite shallower slopes than in the main analysis, the female slope remained steeper than the male slope.

**Reference**

1. Song Z, Sui F, Huang X, Liu MB, Gao W, Weiss JN, *et al.* Population Modeling Approach for Human Cardiac Arrhythmia Risk Prediction. *Circulation: Arrhythmia and Electrophysiology* American Heart Association; **0**:e014249.

2. Orvos P, Kohajda Z, Szlovák J, Gazdag P, Árpádffy-Lovas T, Tóth D, *et al.* Evaluation of Possible Proarrhythmic Potency: Comparison of the Effect of Dofetilide, Cisapride, Sotalol, Terfenadine, and Verapamil on hERG and Native IKr Currents and on Cardiac Action Potential. *Toxicological Sciences* 2019;**168**:365–80.

3. Crumb WJ, Vicente J, Johannesen L, Strauss DG. An evaluation of 30 clinical drugs against the comprehensive in vitro proarrhythmia assay (CiPA) proposed ion channel panel. *Journal of Pharmacological and Toxicological Methods* 2016;**81**:251–62.

4. Darpo B, Karnad DR, Badilini F, Florian J, Garnett CE, Kothari S, *et al.* Are women more susceptible than men to drug-induced QT prolongation? Concentration–QTc modelling in a phase 1 study with oral rac-sotalol. *British Journal of Clinical Pharmacology* 2014;**77**:522–31.

5. Li Z, Dutta S, Sheng J, Tran PN, Wu W, Chang K, *et al.* Improving the In Silico Assessment of Proarrhythmia Risk by Combining hERG (Human Ether-à-go-go-Related Gene) Channel–Drug Binding Kinetics and Multichannel Pharmacology. *Circulation: Arrhythmia and Electrophysiology* American Heart Association; 2017;**10**:e004628.

6. Gaborit N, Varro A, Bouter SL, Szuts V, Escande D, Nattel S, *et al.* Gender-related differences in ion-channel and transporter subunit expression in non-diseased human hearts. *Journal of Molecular and Cellular Cardiology* Elsevier; 2010;**49**:639–46.

7. Holmes M, Wang ZJ, Doste R, Camps J, Martinez-Navarro H, Smith H, *et al.* Sex-specific human electromechanical multiscale in-silico models for virtual therapy evaluation. Physiology; 2025.

8. Yang P-C, Perissinotti LL, López-Redondo F, Wang Y, DeMarco KR, Jeng M-T, *et al.* A multiscale computational modelling approach predicts mechanisms of female sex risk in the setting of arousal-induced arrhythmias. *The Journal of Physiology* 2017;**595**:4695–723.

9. Peirlinck M, Sahli Costabal F, Kuhl E. Sex Differences in Drug-Induced Arrhythmogenesis. *Front Physiol* Frontiers; 2021;**12**.

10. Tomek J, Bueno-Orovio A, Passini E, Zhou X, Minchole A, Britton O, *et al.* Development, calibration, and validation of a novel human ventricular myocyte model in health, disease, and drug block. *eLife* **8**:e48890.

11. Passini E, Britton OJ, Lu HR, Rohrbacher J, Hermans AN, Gallacher DJ, *et al.* Human In Silico Drug Trials Demonstrate Higher Accuracy than Animal Models in Predicting Clinical Pro-Arrhythmic Cardiotoxicity. *Front Physiol* Frontiers; 2017;**8**.

12. Fogli Iseppe A, Ni H, Zhu S, Zhang X, Coppini R, Yang P-C, *et al.* Sex-Specific Classification of Drug-Induced Torsade de Pointes Susceptibility Using Cardiac Simulations and Machine Learning. *Clinical Pharmacology & Therapeutics* 2021;**110**:380–91.

13. Hwang M, Han S, Park MC, Leem CH, Shim EB, Yim D-S. Three-Dimensional Heart Model-Based Screening of Proarrhythmic Potential by in silico Simulation of Action Potential and Electrocardiograms. *Front Physiol* Frontiers; 2019;**10**.

14. Dominguez-Gomez P, Zingaro A, Baldo-Canut L, Balzotti C, Darpo B, Morton C, *et al.* Fast and accurate prediction of drug induced proarrhythmic risk with sex specific cardiac emulators. *npj Digit Med* Nature Publishing Group; 2024;**7**:380.

15. Dasí A, Nagel C, Pope MTB, Wijesurendra RS, Betts TR, Sachetto R, *et al.* In Silico TRials guide optimal stratification of ATrIal FIbrillation patients to Catheter Ablation and pharmacological medicaTION: the i-STRATIFICATION study. *Europace* 2024;**26**:euae150.

16. Louradour J, Hornyik T, De la Cruz A, Hiniesto-Iñigo I, Alerni N, Barbieri M, *et al.* Beneficial action potential duration–shortening effects, but deleterious negative inotropism of IKs-activator docosahexaenoyl glycine in long QT syndrome type 2. *Europace* 2025;**27**:euaf168.
